# Time‐Dependent Predictive Accuracy Metrics in the Context of Interval Censoring and Competing Risks

**DOI:** 10.1002/bimj.70108

**Published:** 2026-01-05

**Authors:** Zhenwei Yang, Dimitris Rizopoulos, Lisa F. Newcomb, Nicole S. Erler

**Affiliations:** ^1^ Department of Epidemiology and Biostatistics Erasmus Medical Center Rotterdam Rotterdam the Netherlands; ^2^ Cancer Prevention Program, Public Health Sciences Fred Hutchinson Cancer Center Seattle Washington USA; ^3^ Julius Center for Health Sciences and Primary Care, University Medical Center Utrecht Utrecht University Utrecht the Netherlands

**Keywords:** accuracy metrics, competing risks, interval censoring, prediction model, time‐varying covariates

## Abstract

Evaluating the performance of a prediction model is a common task in medical statistics. Standard accuracy metrics require the observation of the true outcomes. This is typically not possible in the setting with time‐to‐event outcomes due to censoring. Interval censoring, the presence of time‐varying covariates, and competing risks present additional challenges in obtaining those accuracy metrics. In this study, we propose two methods to deal with interval censoring in a time‐varying competing risk setting: a model‐based approach and the inverse probability of censoring weighting (IPCW) approach, focusing on three key time‐dependent metrics: area under the receiver‐operating characteristic curve, Brier score, and expected predictive cross‐entropy. The evaluation is conducted over a medically relevant time interval of interest, [t,Δt). The model‐based approach includes all subjects in the risk set, using their predicted risks to contribute to the accuracy metrics. In contrast, the IPCW approach only considers the subset of subjects who are known to be event‐free or experience the event within the interval of interest. We performed a simulation study to compare the performance of the two approaches with regard to the three metrics. Furthermore, we demonstrated the three metrics using the two approaches on an example prostate cancer surveillance cohort. Risk predictions were generated from a joint model handling the interval‐censored cancer progression and the competing event, early treatment, and repeatedly measured biomarkers.

## 1 Introduction

Accurate evaluation of a model's predictive accuracy is essential for developing reliable prediction models to guide clinical decisions and public health strategies. Commonly used metrics to evaluate prediction models include the Brier score (to determine how close predictions are to the actual outcomes) (Brier 1950), the area under the receiver operating characteristic (ROC) curve, area under the receiver‐operating characteristic curve (AUC) (to distinguish between low‐ and high‐risk individuals) (Zweig and Campbell 1993), and the expected predictive cross‐entropy (EPCE; to compare the predicted probabilities of events happening and the actual observed events) (Commenges 2015; Commenges et al. 2012). Calculation of these requires researchers to observe the actual outcome (Steyerberg et al. 2010). This is usually the case for continuous and dichotomous outcomes. However, for time‐to‐event data, censoring is common and poses difficulty in determining the occurrence of the outcomes (Leung et al. 1997). The most common type, right censoring, has been extensively investigated. For example, Heagerty et al. (2004) and Wang et al. (2009) proposed to estimate the AUC via the Kaplan–Meier (KM) estimator or an estimator of the bivariate distribution function of the actual outcome and the event time variable. Heagerty and Zheng (2005) demonstrated the calculation of the AUC based on the survival regression models. Harrell's concordance index, as a discrimination metric, deals with right censoring by excluding the patients censored before the time of interest (Harrell et al. 1996).

In settings with interval censoring, the situation is even more complicated because also for patients who are known to have experienced the event of interest during the follow‐up, the exact event time is unknown (Zhang and Sun 2010). Consequently, the ambiguity of the underlying unobserved event times brings challenges in defining cases (patients who experience the event before a timepoint or within a particular time interval) and controls (patients who “survived” up to a timepoint or time interval). The identification of cases and controls is essential in formulating the sensitivity and specificity of the prediction model, based on which the ROC curve is calculated. Tsouprou (2015) described a method to order the event times of any two interval‐censored subjects via a joint density of the two estimated event times when calculating the concordance index. Time‐varying covariates have been drawing greater attention recently in medical applications. The evaluation then needs to be done for a specific time point or a time interval, and the metrics should be a function of time. To accommodate this, time‐dependent evaluation metrics were developed, which allow for the dynamic determination of cases and controls (Bansal and Heagerty 2018). Researchers can then evaluate the model using its predicted risk for an event before a time t+Δt, conditional on covariate information and being event‐free up until a time t. This can be seen as the risk of experiencing an event in a clinically relevant interval [t,t+dt). Last but not least, another common complication is the presence of competing events: patients who are observed with the competing event might still have experienced the (yet unobserved) interval‐censored event. This combination of competing risk and interval censoring is not a rare occurrence in clinical research, for instance, in oncology. Cancer progression or diagnosis is typically not directly observable, and unless all‐cause mortality is the event of interest, death poses a competing risk. This has to be taken into account when obtaining a risk estimate, but it also further complicates the determination of whether the patient is a case or control during model evaluation. Although others have proposed adaptation of the definition of sensitivity being event‐specific (Dey et al. 2020; Saha and Heagerty 2010), the combination of the above‐mentioned difficulties brings forth challenges in the model evaluation.

One notable study by Jacqmin‐Gadda et al. (2016) investigated three approaches to calculate the AUC for a multi‐state model with competing risks and interval censoring, but only considered baseline covariates. The authors proposed two model‐based approaches in which subjects contribute to the sensitivity and specificity in a weighted manner depending on their predicted risk from the model. The other approach corrects for the censoring, typically right censoring, in a nonparametric manner, which uses the inverse probability of censoring weighting (IPCW), whereas the risk probabilities are still derived from a parametric model. Jacqmin‐Gadda et al. (2016) concentrated on marker discrimination, whereas clinical decision‐making often relies on model discrimination (Leening et al. 2014). Except for the AUC as an example of the predictive accuracy metrics, Blanche et al. (2014) proposed the estimation of the Brier score using the IPCW approach in the setting with competing events and time‐varying covariates. They did not, however, consider the setting with interval censoring. Rizopoulos and Taylor (2024) extended the idea of the EPCE proposed by Commenges et al. (2012) and used that as an evaluation metric for a medically relevant time interval in the setting with only time‐varying covariates.

This work aims to extend the formulation of time‐dependent predictive accuracy metrics, specifically the AUC, Brier score, and EPCE, in the context of interval‐censored data with competing risks. We present the adapted accuracy metrics using the Canary Prostate Active Surveillance Study (PASS) (Newcomb et al. 2016). Patients with low‐risk prostate cancer were admitted to active surveillance instead of receiving immediate and typically invasive treatment, and closely monitored by regular biopsies and biomarker measurements (prostate‐specific antigen, PSA). Only when a progression of the cancer is diagnosed, treatment is recommended for these patients. The primary event of interest is prostate cancer progression, which is interval‐censored between a negative biopsy and a positive biopsy. Since the start of treatment means the end of active surveillance, cancer progression while on active surveillance can no longer be observed; thus, the treatment initiation constitutes a competing risk. The example prediction model, based on the motivating data, is a joint model (ICJM) for repeatedly measured PSA and the time to both the primary and competing events (Yang et al. 2025). We explore and extend two methods to evaluate the predictive performance in the context of interval censoring and competing risk: a model‐based approach and the IPCW approach. The former takes advantage of all patients at risk at the beginning of an interval of interest, [t,t+dt), to determine the probabilities of being a case or control for the particular interval and weighs those patients based on their predicted risks from the model or algorithm itself. The latter uses only the subset of the sample for which it is known whether they are a case or a control, and weights this subset to represent the whole sample. We compare the AUC and Brier scores using both approaches and explore the impact of model misspecification as well as different patterns of interval censoring. For the model‐based approach, we also evaluate the EPCE. The corresponding source codes can be found on GitHub (https://github.com/ZhenweiYang96/Predicitve‐accuracy‐metrics). The evaluation metrics are applicable to general statistical models as well as other prediction algorithms, such as machine learning techniques.

The remainder of this paper is presented as follows. The detailed methodology of estimating the three time‐dependent accuracy metrics is demonstrated in Section 2. In Section 3, we present the application of these methods to the ICJM. The results from two simulation studies to investigate the two approaches used on different metrics are displayed in Section 4. At last, there follows a discussion part in Section 5.

## 2 Predictive Accuracy Metrics

### 2.1 Notation

Motivated by the Canary PASS data, we denote δ as the observed event type of patients in the test set, with δ=1 being cancer progression, δ=2 early treatment, and δ=0 right censoring (e.g., due to loss to follow‐up or at the time of data extraction). We distinguish between the true (but unobserved) time of cancer progression ($T^{\mathrm{PRG}*}$), the true time of early treatment initiation ($T^{\mathrm{TRT}*}$), and the censoring time ($T^{\mathrm{CEN}}$). For patients recorded with the interval‐censored event, cancer progression ($T^{\mathrm{PRG}*}<\min\left\{T^{\mathrm{CEN}},T^{\mathrm{TRT}*}\right\}$), it is only known that progression happened between the last progression‐free biopsy (i.e., the observed progression‐free time), $T^{\mathrm{PRG-}}$, and the biopsy time at which progression was detected, $T^{\mathrm{PRG+}}$. For the patients who started early treatment ($T^{\mathrm{TRT}*}<\min\left\{T^{\mathrm{CEN}},T^{\mathrm{PRG+}}\right\}$), progression still might have occurred in the interval between the last progression‐free biopsy and the treatment initiation time $T^{\mathrm{TRT}}=T^{\mathrm{TRT}*}$ but was not detected. For patients who are censored at $T^{\mathrm{CEN}}$, the corresponding progression‐risk interval is between the last progression‐free biopsy and infinity.

We denote the vector of repeated observations of the PSA level with Y. We distinguish the training dataset, used to fit the prediction model, and the test dataset, in which the prediction model is being evaluated. The training set is denoted by $D_{n}$ and includes the longitudinal PSA measurements, event times and other (baseline) covariates of n subjects. We also define an interval of interest for prediction, [t,t+Δt). The quantity of interest is the progression‐specific risk at t+Δt conditional on no event occurring before time t, denoted by $\Pi^{\mathrm{PRG}}\left(t+\Delta t|t\right)$ and can be formulated as

$$\begin{array}{ccc} \Pi^{\mathrm{PRG}}\left(t+\Delta t|t\right) & = & \mathrm{Pr}\left[T^{\mathrm{PRG}*}\leq t+\Delta t,T^{\mathrm{PRG}*}\right.\\ & < & \left.T^{\mathrm{TRT}*}|T^{\mathrm{PRG}*}>t,T^{\mathrm{TRT}*}>t,X\left(t\right),D_{n}\right], \end{array}$$

where X(t) denotes the data of the test set, including the longitudinal PSA measurements up until time t and all other (baseline) covariates used in the prediction. Typically, a subject is considered as a case if their event occurs within the interval of interest, and as a control if the event occurs after that interval. In the test set, the $n_{t}$ patients who have not progressed, been treated, or censored before time t form the risk set and are considered in calculating the evaluation metrics in the following subsections.

### 2.2 Definition of Cases and Controls

**FIGURE 1:**
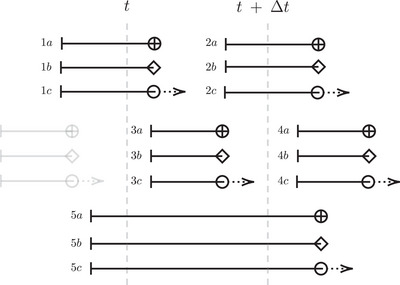
Relative positions of the “risk interval” to the interval of interest [t,t+Δt) (indicated by the dashed vertical lines). Risk intervals start at the time of the last negative biopsy (indicated by ∣) and run until either the detection of progression via a positive biopsy (⊕) or the start of early treatment (◇). In case of right‐censoring (◯), the patient remains at risk for either event (indicated by →). Patients in the grayed out scenarios are excluded from the model evaluation.

The definition and calculation of the AUC and Brier score stems from classifying subjects into cases and controls, that is, whether the subject experiences the event during an interval of interest. Contrary to the case of right‐censored data, for an interval‐censored event, the event time is presented by a risk interval, rather than a single time point. The relative positions of the risk interval and the interval of interest (see Figure 1) bring challenges in clearly distinguishing between patients being a case or a control. The risk interval overlapping with the interval of interest means that the patient has a certain probability of being a case. When the risk interval contains values larger than t+Δt, the patient has a particular probability of being a control.

The patients whose risk interval lies entirely inside the interval of interest and are detected with cancer progression (3a in Figure 1) are known to be cases (“absolute cases”). Likewise, the patients whose risk interval is located entirely after t+Δt (4a, b, and c in Figure 1) are known to be controls (“absolute controls”). Other types of patients are all potential cases and/or controls. Patients whose follow‐up ends before t (shown in grey color in Figure 1) are excluded from the evaluation.

### 2.3 Sensitivity, Specificity, and AUC

To calculate the AUC, the ROC curve has to be determined. This can be done by calculating the sensitivities and 1−specificities corresponding to varying values of the discrimination criterion (Zou et al. 2007). Note that in the following, we calculate the metrics to evaluate the predictions for the primary event of interest, cancer progression.

The progression‐specific sensitivity for a threshold value c, the probability that the predicted risk is larger or equal to c for patients with cancer progression in the interval of interest, [t,t+Δt), can be formulated as

(1) $$\begin{array}{ccc} & & {\text{sen}}^{\mathrm{PRG}}\left(t,\Delta t,c\right)\\ & = & \mathrm{Pr}\{\Pi^{\mathrm{PRG}}\left(t+\Delta t|t\right)\geq c|T^{\mathrm{PRG*}}\geq t,T^{\mathrm{PRG*}}\;<t+\Delta t,T^{\mathrm{PRG*}}\;<T^{\mathrm{TRT*}}\}\\ & = & \frac{\mathrm{Pr}\{\Pi^{\mathrm{PRG}}\left(t+\Delta t|t\right)\geq c,T^{\mathrm{PRG*}}\geq t,T^{\mathrm{PRG*}}\;<t+\Delta t,T^{\mathrm{PRG*}}\;<T^{\mathrm{TRT*}}\}}{\mathrm{Pr}\{T^{\mathrm{PRG*}}\geq t,T^{\mathrm{PRG*}}<t+\Delta t,T^{\mathrm{PRG*}}<T^{\mathrm{TRT*}}\}}, \end{array}$$

where c is a value in [0,1]. Similar definitions can be found in Blanche et al. (2013, 2014). Analogously, following Equation (1) in Blanche et al. (2013), the specificity, used to evaluate the ability to identify the negative instances, is defined as

(2) $$\begin{array}{ccc} & & \text{spe}(t,\Delta t,c)\\ & & =\mathrm{Pr}\{\Pi^{\mathrm{PRG}}\left(t+\Delta t|t\right)\;<c|\min\left(T^{\mathrm{PRG*}},T^{\mathrm{TRT*}}\right)\geq t+\Delta t\}\\ & & =\frac{\mathrm{Pr}\{\Pi^{\mathrm{PRG}}\left(t+\Delta t|t\right)\;<c,\min\left(T^{\mathrm{PRG*}},T_{i}^{\mathrm{TRT*}}\right)\geq t+\Delta t\}}{\mathrm{Pr}\{\min\left(T^{\mathrm{PRG*}},T^{\mathrm{TRT*}}\right)\geq t+\Delta t\}}, \end{array}$$

that is, the probability that the predicted progression‐specific risk for patients who “survive” the interval of interest is lower than the threshold c. In the presence of interval censoring, however, it is often not clear whether a patient is a case, a control, or even had the event before the interval of interest. We propose two methods to deal with this uncertainty when estimating the progression‐specific sensitivity and specificity.

**FIGURE 2:**
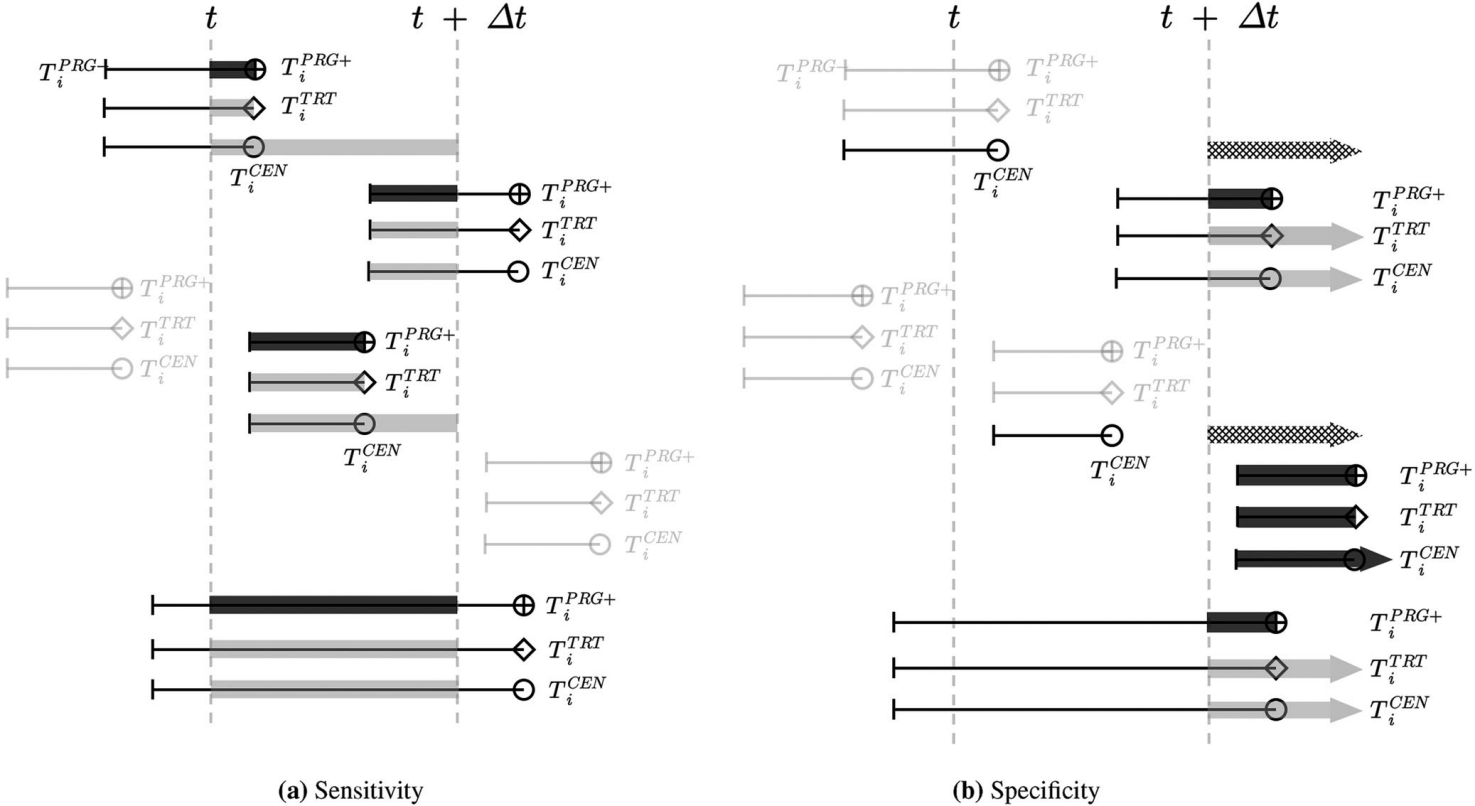
Illustration of the model‐based weights for sensitivity (a) and specificity (b) in calculating AUC and Brier score. The shaded bars represent the time during which the patients contribute to being a case (a) or a control (b). The light‐gray shades are the weights using the predicted progression‐specific risks. The dark shades indicate using the rescaled progression‐specific risks. The crosshatches indicate using the overall survival function. Grayed‐out patient groups are not considered in the calculation.

*Model‐based approach*: In the model‐based approach, all subjects at risk at time t are considered when calculating the sensitivity, but they contribute with different weights, $W_{i}^{M}$, that depend on their estimated probability of experiencing cancer progression during the interval of interest for patient i in the test set. The weights are derived from the cumulative incidence function estimated by the model, and are, thus, named model‐based weights. The estimated model‐based progression‐specific sensitivity can be written as

$$\begin{array}{cc} {\hat{\text{sen}}}_{\mathrm{MODEL}}^{\mathrm{PRG}}\left(t,\Delta t,c\right) & =\frac{\sum_{i}I\left\{\Pi_{i}^{\mathrm{PRG}}\left(t+\Delta t|t\right)\geq c\right\}\times W_{i}^{M}}{\sum_{i}W_{i}^{M}}, \end{array}$$

where I(·) is the indicator function. For different types of patients, the model‐based weights for being a case (visualized in Figure 2a; the numbers in parentheses refer to the numbering used in Figure 1) are specified as

$$\begin{array}{c} W_{i}^{M}=\left\{\begin{array}{cc} \frac{\Pi_{i}^{\mathrm{PRG}}\left(T_{i}^{\mathrm{PRG+}}|T_{i}^{\mathrm{PRG-}}\right)-\Pi_{i}^{\mathrm{PRG}}\left(t|T_{i}^{\mathrm{PRG-}}\right)}{\Pi_{i}^{\mathrm{PRG}}\left(T_{i}^{\mathrm{PRG+}}|T_{i}^{\mathrm{PRG-}}\right)} & \text{if}\;T_{i}^{\mathrm{PRG-}}<t,T_{i}^{\mathrm{PRG+}}>t,T_{i}^{\mathrm{PRG+}}<t+\Delta t,\;\left(1a\right)\\ \Pi_{i}^{\mathrm{PRG}}\left(T_{i}^{\mathrm{TRT}}|T_{i}^{\mathrm{PRG-}}\right)-\Pi_{i}^{\mathrm{PRG}}\left(t|T_{i}^{\mathrm{PRG-}}\right) & \text{if}\;T_{i}^{\mathrm{PRG-}}<t,T_{i}^{\mathrm{TRT}}>t,T_{i}^{\mathrm{TRT}}<t+\Delta t,\;\left(1b\right)\\ \Pi_{i}^{\mathrm{PRG}}\left(t+\Delta t|T_{i}^{\mathrm{PRG-}}\right)-\Pi_{i}^{\mathrm{PRG}}\left(t|T_{i}^{\mathrm{PRG-}}\right) & \text{if}\;T_{i}^{\mathrm{PRG-}}<t,T_{i}^{\mathrm{CEN}}>t,T_{i}^{\mathrm{CEN}}<t+\Delta t.\;\left(1c\right)\\ \frac{\Pi_{i}^{\mathrm{PRG}}\left(t+\Delta t|T_{i}^{\mathrm{PRG-}}\right)}{\Pi_{i}^{\mathrm{PRG}}\left(T_{i}^{\mathrm{PRG+}}|T_{i}^{\mathrm{PRG-}}\right)} & \text{if}\;T_{i}^{\mathrm{PRG-}}>t,T_{i}^{\mathrm{PRG-}}<t+\Delta t,T_{i}^{\mathrm{PRG+}}>t+\Delta t,\;\left(2a\right)\\ \Pi_{i}^{\mathrm{PRG}}\left(t+\Delta t|T_{i}^{\mathrm{PRG-}}\right) & \text{if}\;T_{i}^{\mathrm{PRG-}}>t,T_{i}^{\mathrm{PRG-}}<t+\Delta t,T_{i}^{\mathrm{TRT}}>t+\Delta t,\;\left(2b\right)\\ \Pi_{i}^{\mathrm{PRG}}\left(t+\Delta t|T_{i}^{\mathrm{PRG-}}\right) & \text{if}\;T_{i}^{\mathrm{PRG-}}>t,T_{i}^{\mathrm{PRG-}}<t+\Delta t,T_{i}^{\mathrm{CEN}}>t+\Delta t,\;\left(2c\right)\\ 1 & \text{if}\;T_{i}^{\mathrm{PRG-}}>t,T_{i}^{\mathrm{PRG+}}<t+\Delta t,\;\left(3a\right)\\ \Pi_{i}^{\mathrm{PRG}}\left(T_{i}^{\mathrm{TRT}}|T_{i}^{\mathrm{PRG-}}\right) & \text{if}\;T_{i}^{\mathrm{PRG-}}>t,T_{i}^{\mathrm{TRT}}<t+\Delta t,\;\left(3b\right)\\ \Pi_{i}^{\mathrm{PRG}}\left(t+\Delta t|T_{i}^{\mathrm{PRG-}}\right) & \text{if}\;T_{i}^{\mathrm{PRG-}}>t,T_{i}^{\mathrm{CEN}}<t+\Delta t,\;\left(3c\right)\\ \frac{\Pi_{i}^{\mathrm{PRG}}\left(t+\Delta t|T_{i}^{\mathrm{PRG-}}\right)-\Pi_{i}^{\mathrm{PRG}}\left(t|T_{i}^{\mathrm{PRG-}}\right)}{\Pi_{i}^{\mathrm{PRG}}\left(T_{i}^{\mathrm{PRG+}}|T_{i}^{\mathrm{PRG-}}\right)} & \text{if}\;T_{i}^{\mathrm{PRG-}}<t,T_{i}^{\mathrm{PRG+}}>t+\Delta t,\;\left(5a\right)\\ \Pi_{i}^{\mathrm{PRG}}\left(t+\Delta t|T_{i}^{\mathrm{PRG-}}\right)-\Pi_{i}^{\mathrm{PRG}}\left(t|T_{i}^{\mathrm{PRG-}}\right) & \text{if}\;T_{i}^{\mathrm{PRG-}}<t,T_{i}^{\mathrm{TRT}}>t+\Delta t,\;\left(5b\right)\\ \Pi_{i}^{\mathrm{PRG}}\left(t+\Delta t|T_{i}^{\mathrm{PRG-}}\right)-\Pi_{i}^{\mathrm{PRG}}\left(t|T_{i}^{\mathrm{PRG-}}\right) & \text{if}\;T_{i}^{\mathrm{PRG-}}<t,T_{i}^{\mathrm{CEN}}>t+\Delta t,\;\left(5c\right)\\ 0 & \text{otherwise}\;\left(4a,4b,4c,\;\text{and the grayed-out scenarios}\right), \end{array}\right. \end{array}$$

It is noted that for patient groups 1a, 2a and 5a, since their progression has been detected and must be between the last negative biopsy time $T_{i}^{\mathrm{PRG-}}$ and the positive biopsy time $T_{i}^{\mathrm{PRG+}}$, the weights of being cases are the probabilities of true progression, $T_{i}^{\mathrm{PRG*}}$, in [t,t+Δt) conditional on this true progression observed in $\left(T_{i}^{\mathrm{PRG-}},T_{i}^{\mathrm{PRG+}}\right]$, that is, $\mathrm{Pr}\{T_{i}^{\mathrm{PRG*}}\geq t,T_{i}^{\mathrm{PRG*}}<t+\Delta t|T_{i}^{\mathrm{PRG*}}>T_{i}^{\mathrm{PRG-}},T_{i}^{\mathrm{PRG*}}\leq T_{i}^{\mathrm{PRG+}},T_{i}^{\mathrm{PRG*}}<T_{i}^{\mathrm{TRT*}}\}$. Likewise, the specificity can be estimated correspondingly, by using the model‐based weights (visualized in Figure 2b):

$${\hat{\text{spe}}}_{\mathrm{MODEL}}\left(t,\Delta t,c\right)=\frac{\sum_{i}I\left\{\Pi_{i}^{\mathrm{PRG}}\left(t+\Delta t|t\right)<c\right\}\times {W^{\prime }}_{i}^{M}}{\sum_{i}{W^{\prime }}_{i}^{M}},$$

where the model‐based weights for defining a control are

$$\begin{array}{c} {W^{\prime }}_{i}^{M}=\left\{\begin{array}{cc} S_{i}\left(t+\Delta t|T_{i}^{\mathrm{PRG-}}\right) & \text{if}\;T_{i}^{\mathrm{PRG-}}<t,T_{i}^{\mathrm{CEN}}>t,T_{i}^{\mathrm{CEN}}<t+\Delta t,\;\left(1c\right)\\ \frac{\Pi_{i}^{\mathrm{PRG}}\left(T_{i}^{\mathrm{PRG+}}|T_{i}^{\mathrm{PRG-}}\right)-\Pi_{i}^{\mathrm{PRG}}\left(t+\Delta t|T_{i}^{\mathrm{PRG-}}\right)}{\Pi_{i}^{\mathrm{PRG}}\left(T_{i}^{\mathrm{PRG+}}|T_{i}^{\mathrm{PRG-}}\right)} & \text{if}\;T_{i}^{\mathrm{PRG-}}>t,T_{i}^{\mathrm{PRG-}}<t+\Delta t,T_{i}^{\mathrm{PRG+}}>t+\Delta t,\;\left(2a\right)\\ 1-\Pi_{i}^{\mathrm{PRG}}\left(t+\Delta t|T_{i}^{\mathrm{PRG-}}\right) & \text{if}\;T_{i}^{\mathrm{PRG-}}>t,T_{i}^{\mathrm{PRG-}}<t+\Delta t,T_{i}^{\mathrm{TRT}}>t+\Delta t,\;\left(2b\right)\\ 1-\Pi_{i}^{\mathrm{PRG}}\left(t+\Delta t|T_{i}^{\mathrm{PRG-}}\right) & \text{if}\;T_{i}^{\mathrm{PRG-}}>t,T_{i}^{\mathrm{PRG-}}<t+\Delta t,T_{i}^{\mathrm{CEN}}>t+\Delta t.\;\left(2c\right)\\ S_{i}\left(t+\Delta t|T_{i}^{\mathrm{PRG-}}\right) & \text{if}\;T_{i}^{\mathrm{PRG-}}>t,T_{i}^{\mathrm{CEN}}<t+\Delta t,\;\left(3c\right)\\ 1 & \text{if}\;T_{i}^{\mathrm{PRG-}}>t+\Delta t,\;\left(4a,4b,4c\right)\\ \frac{\Pi_{i}^{\mathrm{PRG}}\left(T_{i}^{\mathrm{PRG+}}|T_{i}^{\mathrm{PRG-}}\right)-\Pi_{i}^{\mathrm{PRG}}\left(t+\Delta t|T_{i}^{\mathrm{PRG-}}\right)}{\Pi_{i}^{\mathrm{PRG}}\left(T_{i}^{\mathrm{PRG+}}|T_{i}^{\mathrm{PRG-}}\right)} & \text{if}\;T_{i}^{\mathrm{PRG-}}<t,T_{i}^{\mathrm{PRG+}}>t+\Delta t,\;\left(5a\right)\\ 1-\Pi_{i}^{\mathrm{PRG}}\left(t+\Delta t|T_{i}^{\mathrm{PRG-}}\right) & \text{if}\;T_{i}^{\mathrm{PRG-}}<t,T_{i}^{\mathrm{TRT}}>t+\Delta t,\;\left(5b\right)\\ 1-\Pi_{i}^{\mathrm{PRG}}\left(t+\Delta t|T_{i}^{\mathrm{PRG-}}\right) & \text{if}\;T_{i}^{\mathrm{PRG-}}<t,T_{i}^{\mathrm{CEN}}>t+\Delta t.\;\left(5c\right)\\ 0 & \text{otherwise}\;\left(1a,1b,3a,3b,\;\text{and the grayed-out scenarios}\right). \end{array}\right. \end{array}$$

The model‐based weights of being controls for the patients who had progression detected and can potentially be a control (i.e., patient group 2a and 5a) are constructed analogously to their counterparts of being cases: the probability of true progression between t+Δt and the first positive biopsy ($T_{i}^{\mathrm{PRG+}}$) conditional on this true progression observed in $\left(T_{i}^{\mathrm{PRG-}},T_{i}^{\mathrm{PRG+}}\right]$. Specifically for patient group 2a, the model‐based weights of being a case and control add up to one, since this patient group can exclusively be classified as either a case or a control. For patient groups 1c and 3c who were censored before the end of the interval of interest (i.e., t+Δt), the model‐based weights for being controls are the probability of the patient not having either of the events before t+Δt conditional on the fact that neither event happened until the last negative biopsy, that is, the conditional overall survival function $S_{i}\left(t+\Delta t|T_{i}^{\mathrm{PRG-}}\right)$. For patients who were censored or treated after t+Δt (i.e., patient groups 2b, 2c, 5b, and 5c), it is known that they did not initiate treatment before t+Δt. Therefore, their weight of being a control is calculated as one minus the conditional progression‐specific risk at t+Δt. A summary and explanation of the specifications of the model‐based weights are presented in Table S1.

*IPCW approach*: Another way to calculate the progression‐specific sensitivity is to utilize only the subset of patients for whom the event is known to be at the interval of interest, that is, the absolute cases (3a in Figure 1), and weigh them to also represent the patients who were censored before experiencing the primary event of interest. These weights are the inverse of the probability of not being censored before time *t*, obtained using the KM estimator. The estimated IPCW‐based progression‐specific sensitivity can be written as:

$$\begin{array}{ccc} & & {\hat{\text{sen}}}_{\mathrm{IPCW}}^{\mathrm{PRG}}\left(t,\Delta t,c\right)\\ & & =\frac{\sum_{i:T_{i}^{\mathrm{PRG-}}\geq t,T_{i}^{\mathrm{PRG+}}<t+\Delta t}I\left\{\Pi_{i}^{\mathrm{PRG}}\left(t+\Delta t|t\right)\geq c\right\}\times W_{i}^{\mathrm{IPCW}}}{\sum_{i:T_{i}^{\mathrm{PRG-}}\geq t,T_{i}^{\mathrm{PRG+}}<t+\Delta t}W_{i}^{\mathrm{IPCW}}}, \end{array}$$

where $W_{i}^{\mathrm{IPCW}}={\left\{G\left(T_{i}^{\mathrm{PRG+}}|t\right)\right\}}^{-1}$ and G(s∣t) is the conditional probability of a patient being censoring‐free at s given being uncensored at t. Furthermore, to estimate the IPCW‐based specificity, the subset of the patients known to be event‐free at t+Δt, that is, the absolute controls (4a, 4b, and 4c in Figure 1), is used:

(3) $${\hat{\text{spe}}}_{\mathrm{IPCW}}\left(t,\Delta t,c\right)=\frac{\sum_{i:T_{i}^{\mathrm{PRG-}}>t+\Delta t}I\left\{\Pi_{i}^{\mathrm{PRG}}\left(t+\Delta t|t\right)<c\right\}\times {W^{\prime }}_{i}^{\mathrm{IPCW}}}{\sum_{i:T_{i}^{\mathrm{PRG-}}>t+\Delta t}{W^{\prime }}_{i}^{\mathrm{IPCW}}}.$$

The corresponding weights are estimated using the KM estimator for being censoring‐free at time t+Δt, to represent the patients censored during the interval of interest. Thus, the weights are the inverse of the conditional probability of being censoring‐free at the end of the interval of interest, ${W^{\prime }}_{i}^{\mathrm{IPCW}}={\left\{G\left(t+\Delta t|t\right)\right\}}^{-1}$. As these weights are uniform for all the controls and will unsurprisingly cancel out, (3) can be simplified to:

$${\hat{\text{spe}}}_{\mathrm{IPCW}}\left(t,\Delta t,c\right)=\frac{\sum_{i:T_{i}^{\mathrm{PRG-}}>t+\Delta t}I\left\{\Pi_{i}^{\mathrm{PRG}}\left(t+\Delta t|t\right)<c\right\}}{\sum_{i:T_{i}^{\mathrm{PRG-}}>t+\Delta t}1}.$$

Using either of the versions of sensitivity and specificity, we can calculate the AUC within [t,t+Δt), AUC(t,Δt), as:

$$\begin{array}{c} \text{AUC}\left(t,\Delta t\right)=\int_{0}^{1}{\text{sen}}^{\mathrm{PRG}}\left(t,\Delta t,c\right)d\left\{1-{\text{spe}}^{\mathrm{PRG}}\left(t,\Delta t,c\right)\right\}. \end{array}$$

### 2.4 Brier score

The Brier score is an evaluation metric that combines discrimination and calibration (Gerds and Schumacher 2006; Steyerberg 2019). It quantifies how close the predicted probabilities are to the actual binary outcomes, with a lower score indicating better model performance. The progression‐specific Brier score has the following formula:

$$\begin{array}{ccc} & & {\text{BS}}^{\mathrm{PRG}}\left(t+\Delta t,t\right)\\ & & =E\left[{\left\{I\left(T_{i}^{\mathrm{PRG*}}<t+\Delta t\right)-\Pi_{i}^{\mathrm{PRG}}\left(t+\Delta t|t\right)\right\}}^{2}|T_{i}^{\left(2\right)}\geq t\right], \end{array}$$

where $T_{i}^{\left(2\right)}$ is $T_{i}^{\mathrm{CEN}}$, $T_{i}^{\mathrm{PRG+}}$, or $T_{i}^{\mathrm{TRT}}$ whichever was observed for patient i. The patient who is a case (i.e., experiencing cancer progression during the interval of interest) is desired to have a probability of predicted risk closer to one, whereas for a control the predicted risk of progression should be low. The Brier score of the test set can be estimated using the model‐based approach as well as the IPCW approach, using the same subsets of patients and weights as for the AUC.

*Model‐based approach:* Utilizing the set of patients at risk of cancer progression at time t and the model‐based weights given in Section 2.3, the Brier score can be estimated by:

$$\begin{array}{ccc} {\hat{\text{BS}}}_{\mathrm{MODEL}}^{\mathrm{PRG}}\left(t+\Delta t,t\right) & = & \frac{1}{n_{t}}\sum_{i:T_{i}^{\left(2\right)}\geq t}\left[{\left\{1-\Pi_{i}^{\mathrm{PRG}}\left(t+\Delta t|t\right)\right\}}^{2}\right.\\ & & \times \left.W_{i}^{M}+{\left\{0-\Pi_{i}^{\mathrm{PRG}}\left(t+\Delta t|t\right)\right\}}^{2}\times {W^{\prime }}_{i}^{M}\right], \end{array}$$

where $n_{t}$ is the number of patients who have not been detected with progression, been treated, or censored until time t.

*IPCW approach*: Analogously, using the IPC weights (as specified in Section 2.3), we have:

$$\begin{array}{ccc} & & {\hat{\text{BS}}}_{\mathrm{IPCW}}^{\mathrm{PRG}}\left(t+\Delta t,t\right)\\ & & =\frac{1}{n_{t}}\left[\sum_{i:T_{i}^{\mathrm{PRG-}}\geq t,T_{i}^{\mathrm{PRG+}}<t+\Delta t}{\left\{1-\Pi_{i}^{\mathrm{PRG}}\left(t+\Delta t|t\right)\right\}}^{2}\times W_{i}^{\mathrm{IPCW}}\right.\\ & & \left.+\sum_{i:T_{i}^{\mathrm{PRG-}}>t+\Delta t}{\left\{0-\Pi_{i}^{\mathrm{PRG}}\left(t+\Delta t|t\right)\right\}}^{2}\times {W^{\prime }}_{i}^{\mathrm{IPCW}}\right]. \end{array}$$

For calculating the first factor, only the subjects detected with cancer progression within the interval of interest are used whereas for the second factor only the subjects who are event‐free until t+Δt contribute via their weights.

### 2.5 Expected Predictive Cross‐Entropy

The EPCE is an evaluation quantity from information theory, which describes the expected value of the cross‐entropy between the true and predicted risk distributions (Commenges et al. 2012). In our setting, we focus on the density of cancer progression risk. The progression‐specific EPCE has the following form:

$$\begin{array}{ccc} {\text{EPCE}}^{\mathrm{PRG}}\left(t+\Delta t,t\right) & & =E\left\{-\log\left[p\left\{T_{i}^{\mathrm{PRG}*}|T_{i}^{\mathrm{PRG}*}\geq t,T_{i}^{\mathrm{PRG}*}\right.\right.\right.\\ & & <\left.\left.\left.t+\Delta t,T_{i}^{\mathrm{PRG}*}<T_{i}^{\mathrm{TRT}*},Y_{i}\left(t\right),D_{n}\right\}\right]\right\}. \end{array}$$

The EPCE can be estimated by:

$$\begin{array}{ccc} {\hat{\text{EPCE}}}^{\mathrm{PRG}}\left(t+\Delta t,t\right) & & =\frac{1}{n_{t}}\sum_{i:T_{i}^{\left(2\right)}\geq t}-\log\left[p\{{\tilde{T}}_{i}^{\left(1\right)},{\tilde{T}}_{i}^{\left(2\right)},{\tilde{\delta }}_{i}^{\left(1\right)},{\tilde{\delta }}_{i}^{\left(2\right)}|T_{i}^{\mathrm{PRG}*}\right.\\ & & \left.\geq t,T_{i}^{\mathrm{PRG}*}<T_{i}^{\mathrm{TRT}*},Y_{i}\left(t\right),D_{n}\}\right], \end{array}$$

where ${\tilde{T}}_{i}^{\left(1\right)}=\max\left(T_{i}^{\mathrm{PRG-}},t\right)$,

$$\begin{array}{c} {\tilde{T}}_{i}^{\left(2\right)}=\left\{\begin{array}{cc} \min(T_{i}^{\mathrm{PRG+}},t+\Delta t), & \delta_{i}=1,\\ \min(T_{i}^{\mathrm{TRT}},t+\Delta t), & \delta_{i}=2,\\ t+\Delta t, & \delta_{i}=0, \end{array}\right. \end{array}$$

${\tilde{\delta }}_{i}^{\left(1\right)}=I\left(T_{i}^{\mathrm{PRG-}}\leq t+\Delta t,T_{i}^{\left(2\right)}\geq t\right)$, and ${\tilde{\delta }}_{i}^{\left(2\right)}=I\left(T_{i}^{\mathrm{PRG-}}\geq t+\Delta t,\delta_{i}=0\right)$. The term in the log function can be estimated by:

$$\begin{array}{ccc} & & p\{{\tilde{T}}_{i}^{\left(1\right)},{\tilde{T}}_{i}^{\left(2\right)},{\tilde{\delta }}_{i}^{\left(1\right)},{\tilde{\delta }}_{i}^{\left(2\right)}|T_{i}^{\mathrm{PRG}*}\geq t,T_{i}^{\mathrm{PRG}*}<T_{i}^{\mathrm{TRT}*},Y_{i}\left(t\right),D_{n}\}\\ & & \;=\log\left[{\tilde{\delta }}_{i}^{\left(1\right)}F_{1}+{\tilde{\delta }}_{i}^{\left(2\right)}F_{2}\right], \end{array}$$

where the first factor, $F_{1}=\int_{{\tilde{T}}_{i}^{\left(1\right)}}^{{\tilde{T}}_{i}^{\left(2\right)}}\mathrm{Pr}\left\{{\tilde{T}}_{i}^{\left(1\right)}\leq T_{i}^{\mathrm{PRG}*}<s|T_{i}^{\mathrm{PRG}*}\geq t,T_{i}^{\mathrm{PRG}*}<T_{i}^{\mathrm{TRT}*},Y_{i}\left(t\right),D_{n}\right\}ds$, is the cumulative incidence function of progression over ${\tilde{T}}_{i}^{\left(1\right)}$ and ${\tilde{T}}_{i}^{\left(2\right)}$, and the second factor, $F_{2}=\frac{\mathrm{Pr}\{T_{i}^{*}\geq t+\Delta t|Y_{i}\left(t\right),D_{n}\}}{\mathrm{Pr}\{T_{i}^{*}\geq t|Y_{i}\left(t\right),D_{n}\}}$, is the overall survival probability of the patient being event‐free at t+Δt conditional on that he did not experience either of the events until t.

## 3 Application to the ICJM

To illustrate the practical application of these methods, we analyze a joint model that can handle interval censoring and competing risks fitted on data from the Canary Prostate Active Surveillance Study. This study focuses on the progression of prostate cancer to a stage necessitating intervention, with progression detected through interval‐censored biopsies. Additionally, the initiation of early treatment, which occurs before the cancer reaches a critical stage, is considered a competing risk. The patients were examined with biopsies according to the PASS schedules (at 12, 24 months, and biennially afterwards) to detect the primary event of interest, cancer progression, resulting in interval censoring. Around 10% of the patients left AS for treatment before cancer progression was detected, constituting the competing event (Newcomb et al. 2016). In addition, the repeatedly measured biomarker, PSA, is a time‐varying predictor for the events of interest. We developed the ICJM 1 in Yang et al. (2025) from the Canary PASS data (more details can be found in Supporting Information Appendix 2). The repeatedly measured PSA levels, for patient j in the training set, were modeled in the longitudinal submodel of the ICJM, a mixed‐effects model,

$$\begin{array}{ccc} \log_{2}\left\{{\text{PSA}}_{j}\left(t\right)+1\right\} & = & \beta_{0}+u_{0j}+\sum_{p=1}^{3}\left(\beta_{p}+u_{pj}\right)C_{j}^{\left(p\right)}\left(t\right)\\ & & +\;\beta_{4}\left({\text{Age}}_{j}-62\right)+\varepsilon_{j}\left(t\right),\;u_{j}∼N\left(0,\Omega \right), \end{array}$$

where C(·) is the design matrix for the natural cubic splines, ${\text{Age}}_{j}$ refers to the patient's age at the start of active surveillance and $\beta ={\left(\beta_{0},\cdots ,\beta_{4}\right)}^{⊤}$ is a vector of corresponding regression coefficients. The error terms $\varepsilon_{j}\left(t\right)$ were assumed to follow a Student's *t*‐distribution with three degrees of freedom, and the random effects, $u_{j}={\left\{u_{0i},u_{1i},u_{2i},u_{3i}\right\}}^{⊤}$, were assumed to be normally distributed with a mean of zero and an unstructured variance–covariance matrix Ω. The expected values of $\log_{2}\left\{{\text{PSA}}_{j}\left(t\right)+1\right\}$, which we denote by $m_{j}\left(t\right)$, were then incorporated into a survival submodel using two functional forms:

$$\begin{array}{ccc} h_{j}^{\left(k\right)}\left\{t|M_{j}\left(t\right),{\text{PSAD}}_{j}\right\} & = & h_{0}^{\left(k\right)}\left(t\right)\exp\left[\gamma_{k}^{⊤}{\text{PSAD}}_{j}+\alpha_{1k}m_{j}\left(t\right)\right.\\ & & +\left.\alpha_{2k}\left\{m_{j}\left(t\right)-m_{j}\left(t-1\right)\right\}\right], \end{array}$$

where the hazard of the jth patient in the training set for event k (k∈K={PRG,TRT}, where PRG stands for cancer progression and TRT for early treatment) at time t is denoted as $h_{j}^{\left(k\right)}\left(t\right)$. The vector $M_{j}\left(t\right)=\left\{m_{j}\left(s\right);0\leq s<t\right\}$, contains the estimated PSA trajectories until t, with the corresponding regression coefficient $\alpha_{k}$ for the functional forms of estimated PSA levels, and ${\text{PSAD}}_{j}$ is the baseline PSA density (calculated as the baseline PSA level divided by the baseline prostate gland's volume), with corresponding regression coefficient $\gamma_{k}$. The model included two functional forms of the estimated PSA levels: the estimated PSA level at t, $m_{j}\left(t\right)$, and the yearly change in the estimated PSA, $m_{j}\left(t\right)-m_{j}\left(t-1\right)$. The event k‐specific baseline hazard $h_{0}^{\left(k\right)}\left(t\right)$ was specified with the penalized B‐splines:

$$\begin{array}{c} \log h_{0}^{\left(k\right)}\left(t\right)=\gamma_{k,h_{0},0}+\sum_{a=1}^{A}\gamma_{k,h_{0},a}G_{a}\left(t,\xi \right), \end{array}$$

where $G_{a}\left(t,\xi \right)$ is the ath basis function of a B‐splines with knots $\xi_{1},\cdots ,\xi_{A}$. The number of knots was chosen to be 11, and the penalized coefficients for the basis function are denoted by $\gamma_{k,h_{0}}$. The ICJM was estimated in the Bayesian framework. We evaluated the predictive performance of the ICJM for the interval of interest, (t,t+Δt]=(1,4], that is, using covariate information up until year 1. The evaluation was performed internally on the Canary PASS data using fivefold cross‐validation. A total of 812 (out of 833) subjects at risk in year one were included in the evaluation. During the interval of interest, 50 subjects started the competing event, early treatment. The model‐based approach resulted in an AUC of 0.64, while the AUC estimated using the IPCW approach was higher, namely 0.68. Also in the calculation of the Brier score, the model‐based approach estimated a less favorable performance than the IPCW approach, namely 0.17 versus 0.07. The model's estimated EPCE was −0.72.

## 4 Simulation

### 4.1 Simulation Setting

As the results of the application in Section 3 show, the model‐based and IPCW approach can result in relatively different estimates of AUC and Brier score. To investigate the performance of the two methods in a more controlled setting, we conducted two simulation studies. In both studies, the accuracy metrics were calculated using the longitudinal information (i.e., PSA) until year one in the test set and evaluating the performance up until year four, that is, the interval of interest being [1,4). Two hundred datasets were simulated based on the ICJM handling interval censoring of biopsies and competing risk of early treatment described in Section 3, previously developed in Yang et al. (2025), and the PASS biopsy schedule (Newcomb et al. 2016), each with 300 subjects. In the 200 datasets, there are, on average, 22% patients who were observed with cancer progression and 9% who initiated early treatment.

### 4.2 Comparison Between Model Specifications

Since the model‐based approach reuses the prediction model for its evaluation, model misspecification might lead to overestimation of the model's predictive performance. Therefore, in this simulation, we aim to explore the influence of model misspecification on the accuracy metrics from both approaches. Three models, including the correctly specified model and two misspecified models (i.e., ignoring one baseline covariate in the survival submodel and wrongly assuming a linear evolution for the PSA trajectory), were fitted on each dataset (as the training set). Each fitted model was then evaluated on another simulated dataset (as the test set in which we utilize the PSA information until year one for predictions). As a reference, we also calculated AUC, Brier score, and EPCE, based on the true event times, that is, for the ideal scenario where there is no censoring, and it is, thus, known whether a patient is a case or a control. It is noted that the reference metrics were calculated using the same risk estimates from the corresponding model when compared to the model‐based and IPCW approach. Furthermore, we also presented a naive calculation of AUC and Brier score, that is, ignoring the interval censoring essence of cancer progression and using the observed progression time to determine a case or control.

**FIGURE 3:**
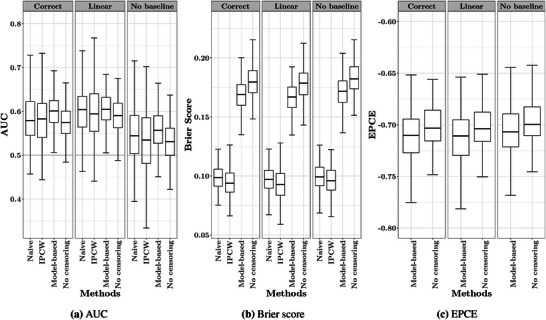
The model‐based, IPCW and naive (ignoring interval censoring) estimates of the AUC (a) and Brier score (b), and the model‐based estimates of the EPCE (c) from the correctly specified model, the model with a linear PSA trajectory, and the model ignoring the baseline covariate (PSA density), compared to the corresponding ideal scenario when there is no censoring.

The main results from the simulation study are shown in Figure 3 and Table 1. Comparison of the AUC between the three models for the ideal scenario without censoring (Figure 3a) shows that misspecification of the effect of time on PSA as linear had no relevant effect on the model's performance, whereas omitting the baseline covariate considerably worsened the discriminative ability. Simply ignoring interval censoring resulted in, on average, relatively small differences in the AUC compared to the ideal scenario in which the exact event times are known. However, the variability in this approach was larger than in the model‐based approach. The naive approach performed worse than the model‐based approach but was comparable to the IPCW approach in estimating the Brier score for all three models whether misspecified or not. The IPCW estimates of the AUC generally had greater variability, whereas the median estimates across all three models were closer to the reference AUC than the median of the model‐based estimates. The model‐based approach tended to overestimate the AUC. Misspecification of the model by omitting the baseline covariate resulted in a larger root mean square error (RMSE) for the model‐based approach. However, the variability of the model‐based AUC across simulations was not inflated. Analogous to the AUC, the model‐based Brier scores for all three models had less variability but were, on average, closer to the reference Brier score than the one from the IPCW approach. Only the model‐based approach generated estimates of the Brier score that were similar to the ideal scenario for all three models. The model‐based approach also had the lowest RMSE in estimating both the AUC and Brier scores for all three models, approximately 38% better for the AUC and 83% better for the Brier score compared to the IPCW approach, and approximately 24% better for the AUC and 82% better for the Brier score compared to the naive approach. The model‐based estimates of the EPCE for the three models were comparable to the reference EPCE (see Figure 3c). The reference EPCE also did not vary much among different models.

**TABLE 1:** The root mean square error of the model‐based, IPCW, and naive (ignoring interval censoring) estimates of the AUC (a) and Brier score (b) from the correctly specified model, the model with a PSA trajectory, and the model ignoring the baseline covariate (PSA density), to the corresponding reference metrics without censoring.

(a) AUC				(b) Brier score			
	Model‐based	IPCW	Naive		Model‐based	IPCW	Naive
Correctly specified	0.036	0.056	0.045		0.015	0.087	0.083
Linear	0.029	0.054	0.043		0.015	0.086	0.082
No baseline covariate	0.040	0.058	0.051		0.015	0.088	0.085

### 4.3 Comparison Between Biopsy Frequencies

**FIGURE 4:**
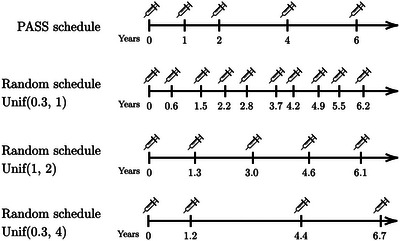
The visualizations of one example series of schedules in the second simulation.

The uncertainty due to interval censoring is closely related to the frequency of examination, in our case, the biopsies. With more recurrent biopsies, the true event times are restricted to shorter intervals and, thus, are expected to increase the predictive performance. Therefore, this second simulation study aims to explore the potential effect of the information intensity from the interval censoring on different approaches of estimating the AUC and Brier score. Again, we simulated the datasets based on the ICJM 1 but applied four different biopsy schedules, namely the PASS schedule, and three schedules with random biopsy intervals, uniformly distributed between 0.3 and 1 year, 1 and 2 years, and 0.3 and 4 years, respectively (see examples for different scenarios in Figure 4). For simplicity, we only compare the estimates of the AUC and Brier score from the correctly specified model in this case.

**FIGURE 5:**
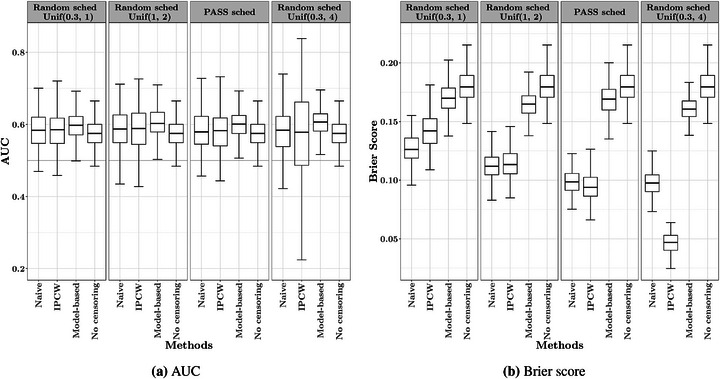
The model‐based, IPCW and naive (ignoring interval censoring) estimates of the AUC (a) and Brier score (b) from the correctly specified model, in the context of a PASS biopsy schedule, a random schedule from 0.3 to 1 year, a random schedule from 1 to 2 years, and a random schedule from 0.3 to 4 years, compared to the corresponding ideal scenario when there is no censoring.

The estimated AUC and Brier scores from three approaches and different biopsy frequencies are depicted in Figure 5, and the corresponding calculated RMSE are listed in Table 2. With shorter biopsy intervals (i.e., more frequent biopsies), the IPCW estimates of the AUC tended to have lower variability, whereas the variability in the model‐based approach was less impacted by the biopsy schedules. This inflation of the variability with less frequent biopsies was observed in the naive approach as well, but was less obvious than in the IPCW approach. With larger biopsy intervals, that is, fewer biopsies, the bias in the model‐based AUC estimates increased while the median of the IPCW estimates was always closer to that of the ideal AUC and remained more stable across different biopsy schedules. All three approaches had similar RMSE of the AUC estimates in the most frequent biopsy schedule. The model‐based approach, on average for the four schedules, improved the RMSE of AUC estimates by 38% relative to the IPCW approach and 10% relative to the naive approach. The model‐based Brier scores were much closer to those from the ideal scenario without censoring and were more robust to lower biopsy frequencies. The Brier score estimates from the IPCW and naive approach differed a lot from those from the ideal scenario, especially with larger biopsy intervals. This tendency was much more manifest in the IPCW Brier scores. Regarding the RMSE of the Brier score, the model‐based approach was on average 79% better than the IPCW approach and 76% better than the naive approach. The variability of all Brier score estimates was robust to the biopsy schedules.

**TABLE 2:** The root mean square error of the model‐based, IPCW, and naive (interval censoring) estimates of the AUC (a) and Brier score (b) from the correctly specified model, in the context of a PASS biopsy schedule, a random schedule from 0.3 to 1 year, a random schedule from 1 to 2 years, and a random schedule from 0.3 to 4 years, to the corresponding reference metrics without censoring.

(a) AUC				(b) Brier score			
	Model‐based	IPCW	Naive		Model‐based	IPCW	Naive
Random schedule Unif(0.3,1)	0.038	0.040	0.039		0.013	0.039	0.055
Random schedule Unif(1,2)	0.045	0.054	0.046		0.019	0.067	0.070
PASS schedule	0.036	0.056	0.045		0.015	0.087	0.083
Random schedule Unif(0.3,4)	0.047	0.118	0.054		0.023	0.135	0.084

## 5 Discussion

In this study, we presented two approaches to estimate commonly used time‐dependent predictive accuracy metrics, the AUC, Brier score, and EPCE, for a prediction model or algorithm in a setting with competing risks and interval censoring: a model‐based and an IPCW approach. The two approaches follow different strategies to deal with the uncertainty about a patient's true event status at a particular time of interest due to censoring. The model‐based approach uses all subjects in the risk set for evaluation and weighs their contributions to the estimation based on the estimated probability of experiencing the event (i.e., being a case) or “surviving” the interval of interest (i.e., being a control). The IPCW approach only utilizes those subjects in the estimation that are known to be a case or a control. Our simulation study showed that the IPCW estimates typically have greater variability but are more robust to model misspecification. The model‐based approach was more sensitive to the model misspecification but more robust to the frequency of the examinations (resulting in interval censoring). A possible reason is that for sparser biopsy schedules, not only the uncertainty about the true event time is larger, but, as a result, fewer patients will be included in the IPCW approach since it is less likely that the risk interval between a negative and positive biopsy is fully contained in the interval of interest. In most of the scenarios, especially the Brier score and the combination of precision and variability (i.e., RMSE), the model‐based approach outperformed the IPCW approach. The EPCE can only be calculated for the model‐based approach. Even with interval censoring and competing risks, the model‐based EPCE can recover the reference EPCE (i.e., in the ideal scenario without any censoring).

One notable point when using time‐dependent accuracy metrics is the choice of the interval of interest. For the IPCW approach, it is necessary to have both cases (i.e., the interval between a negative and a positive examination must be fully contained in the interval of interest) and controls (i.e., the negative examination is after the end of the interval of interest). Therefore, the length of the interval of interest has to be longer than the minimal length of the examination interval. In the case of a fixed schedule like the Canary PASS, the timing of the biopsies should also be taken into account. Furthermore, the model is expected to have better predictive performance for later intervals of interest since more longitudinal information is used in that case. On the other hand, when the interval of interest is late in the follow‐up, there will be few(er) “absolute controls.” In our first simulation study, it was noticed that the linear model was highly comparable to the correctly specified model. This is because the predictions were based on the PSA measurements in the first year, and the simulated (overall nonlinear) PSA trajectories were relatively linear during the first year (see Figure S1).

As with all statistical methods, the two approaches presented here have strengths and limitations. The model‐based approach typically has less variability and performs much better than the IPCW approach in estimating the Brier score. In addition, the model‐based approach is capable of handling informative censoring that depends on the history of the longitudinal outcome(s), which the IPCW approach does not directly accommodate (Rizopoulos and Taylor 2024). However, it tends to be overly optimistic since the estimated risk probabilities from the model itself are used in its evaluation, making this approach's performance more dependent on the correct model specification. On the other hand, the IPCW‐based AUC estimates were less biased, but this approach has the disadvantage of a potentially large information loss, particularly in the setting with interval censoring where there may be only a few “absolute cases” and/or “absolute controls.” This issue is exacerbated when the examinations are infrequent and/or the interval of interest is too long. Moreover, the IPCW approach persistently underestimating the Brier score might be related to the IPC weights being designed to handle right censoring and failing to correct the uncertainty brought from interval censoring. Future studies are required to address this limitation. The IPCW approach requires an additional assumption that the timing of the examination (in our case, the biopsies) is not dependent on any covariate information or previously observed repeated measurements. This assumption is not necessary for the model‐based approach. In our application, the assumption holds, as the biopsy schedule was predefined in the Canary PASS protocol. Other than the nonparametric way to calculate IPC weights, using semiparametric models including important biomarker information as covariates is an alternative to calculate the patient‐specific IPC weights (Blanche et al. 2013).

Our evaluation of the investigated methods has some limitations. The simulation study was set up to evaluate the performance of the two methods in our own real‐world application. In that setting, our choices were restricted by the fact that most of the detected progression occurred around year three and that biopsies around the most cancer progression were performed in years 1, 2, and 4. The interval of interest should start earlier than year two or end later than year 4 to ensure the possibility of having “absolute cases.” It is expected to improve the predictive performance if the interval of interest could be chosen to contain enough “absolute cases” and “absolute controls” and simultaneously take advantage of enough longitudinal information. However, we added the second simulation to explore how these results can be generalized to other settings in which the intervals between examinations may be different. Moreover, in this study, we assumed the sensitivity of biopsies to be perfect, meaning that the underlying cancer progression can only occur between a negative and a positive biopsy. Nevertheless, in real practice, there exist many periodic examinations with low sensitivity, indicating that the underlying event(s) might have occurred even before the negative examination. This brings an extra challenge to the underlying timing of interval‐censored event(s), and consequently leads to difficulty in defining cases and controls. Our previous study solved this problem in the model estimation phase (Yang et al. 2024). Similarly, the corresponding weights in the accuracy metrics could be calculated as a weighted sum of the event‐specific risks in each related examination period multiplied by the misclassification probability determined by a prespecified test sensitivity.

To conclude, the model‐based approach is a reliable method in terms of bias and variability for evaluating prediction models with time‐varying covariates, competing risks, and interval censoring, especially when interval censoring results in losing the information of a subject being a case or control that is essential in the IPCW approach.

## Conflicts of Interest

The authors declare no conflicts of interest.

## Open Research Badges

This article has earned an Open Data badge for making publicly available the digitally‐shareable data necessary to reproduce the reported results. The data is available in the Supporting Information section.

This article has earned an open data badge “**Reproducible Research**” for making publicly available the code necessary to reproduce the reported results. “The results reported in this article could fully be reproduced.”

## Supporting information


**Supporting File:** bimj70108‐sup‐0001‐SuppMat.pdf.

**Supporting File:** bimj70108‐sup‐0002‐Datacode.zip.

## Data Availability

The data that support the findings of this study are available on request from the corresponding author. The data are not publicly available due to privacy or ethical restrictions.
