# Supplementary material for: Time‐Dependent Predictive Accuracy Metrics in the Context of Interval Censoring and Competing Risks
Source: Biom J. 2026 Jan 5;68(1):e70108. doi: 10.1002/bimj.70108 (PMC12766878; doi:10.1002/bimj.70108)
Supplement: Supplementary file 1 — Supporting File: bimj70108‐sup‐0001‐SuppMat.pdf. [file BIMJ-68-e70108-s002.pdf]

Supporting material - Time-dependent Predictive Accuracy Metrics in the Context of Interval Censoring and Competing Risks

Zhenwei Yang[1], Dimitris Rizopoulos[1], Lisa F. Newcomb[2], and Nicole S. Erler[1,3]

[1]Department of Epidemiology and Biostatistics, Erasmus Medical Center Rotterdam
[2]Fred Hutchinson Cancer Center, Cancer Prevention Program, Public Health Sciences, Seattle, Washington
[3]Julius Center for Health Sciences and Primary Care, University Medical Center Utrecht, Utrecht University

Web Appendix 1 Canary PASS Data

Web Appendix 1.1 Rationale of the competing risk, early treatment

AS is acknowledged as a way to reduce overtreatment for prostate cancer. Low-risk cancer patients are admitted to AS where they are monitored by regular biopsies and (more frequent) blood testing. The (often invasive) treatment, such as radiation or prostatectomy, is deferred until cancer progression (to a Gleason score ≥ 7) is detected. However, not everyone strictly follows this protocol. Some patients initiate treatment earlier, without cancer progression being detected, commonly due to personal reasons, such as anxiety of carrying cancer that might progress, change in preference between regular biopsies or the curative treatment.[Beckmann et al., 2021, Tosoian et al., 2016] This early treatment is considered as the competing event in this study as cancer progression can no longer be observed once early treatment is initiated.

Web Appendix 2 The Interval-censored Cause-specific Joint model (ICJM)

The ICJM mentioned in the manuscript handles the competing risk from early treatment and interval censoring of cancer progression due to periodic biopsies via the likelihood. The likelihood of the survival

part for the patient j in the training set can be written as:

$$p(\boldsymbol{T}_j, \delta_j \mid \boldsymbol{u}_j, \boldsymbol{\theta}) = \left[\exp\left\{-\int_0^{T_j^{\text{PRG-}}} h_j^{(\text{PRG})}(\nu)d\nu - \int_0^{T_j^{\text{CEN}}} h_j^{(\text{TRT})}(\nu)d\nu\right\}\right]^{I(\delta_j=0)}$$
$$\times \left[\int_{T_j^{\text{PRG-}}}^{T_j^{\text{PRG+}}} h_j^{(\text{PRG})}(s)\exp\left\{-\int_0^{s} h_j^{(\text{PRG})}(\nu)d\nu - \int_0^{T_j^{\text{PRG+}}} h_j^{(\text{TRT})}(\nu)d\nu\right\}ds\right]^{I(\delta_j=1)}$$
$$\times \left[h_j^{(\text{TRT})}(T_j^{\text{TRT}})\exp\left\{-\int_0^{T_j^{\text{PRG-}}} h_j^{(\text{PRG})}(\nu)d\nu - \int_0^{T_j^{\text{TRT}}} h_j^{(\text{TRT})}(\nu)d\nu\right\}\right]^{I(\delta_j=2)},$$

where $\boldsymbol{T}_j$ is the time vector for patient j that may include the last negative biopsy time $T_j^{\text{PRG-}}$, and censoring time T_j^{CEN}, first positive biopsy time (cancer progression detection time) $T_j^{\text{PRG+}}$, or treatment initiation time T_j^{TRT}; $\delta_j = \{0, 1, 2\}$ is the observed event indicator with 0 for the censored patients, 1 for the patients detected with cancer progression and 2 for the patients who started early treatment; $h_j^{(\text{PRG})}(\cdot)$ and $h_j^{(\text{PRG})}(\cdot)$ are the progression- and treatment-specific instantaneous hazards. The first factor (for $\delta_j = 0$), is the probability of not having experienced any event up until the respective event-free times. Since it is only known that cancer progression did not happen until the last biopsy, patients contribute to the "overall survival" part of the likelihood only until their event-specific event-free times, the last biopsy time $T_j^{\text{PRG-}}$ and censoring time (T_j^{CEN}). The second factor (for $\delta_j = 1$) models the probability of patients having progression in the interval between the last progression-free biopsy, $T_j^{\text{PRG-}}$, and the biopsy at which progression was detected, $T_j^{\text{PRG+}}$. For those patients the "overall survival" part includes the probability that patients are progression-free until time s, where s ranges over the interval $(T_j^{\text{PRG-}}, T_j^{\text{PRG+}}]$, as well as the probability that the patient did not initiate early treatment before the detection of progression, i.e., until $T_j^{\text{PRG+}}$. The third factor (for $\delta_j = 2$) uses the standard cause-specific cumulative incidence function in the "overall survival" part to formulate the probability of patients starting early treatment at $T_j^{\text{TRT+}}$, conditional on patients not having progressed until the last biopsy (i.e., progression-free time) $T_j^{\text{PRG-}}$. More details can be found in Yang et al. [2025].

Web Appendix 2.1 Prediction

Using the baseline covariates and time-varying biomarker information up until time t, we can calculate the probability of the new patient i (in the test dataset) experience the event of interest (in this case, cancer progression) before $t+\Delta t$ conditional on that they do not have either event until t, $\Pi_i^{\text{PRG}}(t+\Delta t \mid t)$:

$$\Pi_i^{\text{PRG}}(t+\Delta t \mid t) = \Pr\left[T_i^{\text{PRG}*} \leq t+\Delta t, T_i^{\text{PRG}*} < T_i^{\text{TRT}*} \mid T_i^{\text{PRG}*} > t, T_i^{\text{TRT}*} > t, \boldsymbol{\mathcal{X}}_i(t), \boldsymbol{\mathcal{D}}_n\right]$$
$$= \int\int \Pr\left\{T_i^{\text{PRG}*} \leq t+\Delta t, T_i^{\text{PRG}*} < T_i^{\text{TRT}*} \mid T_i^{\text{PRG}*} > t, T_i^{\text{TRT}*} > t, \boldsymbol{u}_i, \boldsymbol{\theta}\right\}$$
$$p\left\{\boldsymbol{u}_i \mid T_i^{\text{PRG}*} > t, T_i^{\text{TRT}*} > t, \boldsymbol{\mathcal{X}}_i(t), \boldsymbol{\theta}\right\}$$
$$p(\boldsymbol{\theta} \mid \boldsymbol{\mathcal{D}}_n)d\boldsymbol{u}_i d\boldsymbol{\theta},$$

where the first term inside the integral can be rewritten based on the Bayes rule as

$$
\begin{aligned}
&\Pr\left\{T_i^{\text{PRG}*} \le t+\Delta t, T_i^{\text{PRG}*} < T_i^{\text{TRT}*} \mid T_i^{\text{PRG}*} > t, T_i^{\text{TRT}*} > t, \boldsymbol{u}_i, \boldsymbol{\theta}\right\} \\
&= \frac{\Pr\{t < T_i^{\text{PRG}*} \le t+\Delta t, \max(T_i^{\text{PRG}*}, t) < T_i^{\text{TRT}*} \mid \boldsymbol{u}_i, \boldsymbol{\theta}\}}{\Pr\{T_i^{\text{PRG}*} > t, T_i^{\text{TRT}*} > t \mid \boldsymbol{u}_i, \boldsymbol{\theta}\}} \\
&= \frac{\displaystyle\int_t^{t+\Delta t} h_i^{(\text{PRG})}(\nu)\exp\left[-\int_0^{\nu} h_i^{(\text{PRG})}(s)ds - \int_0^{\nu} h_i^{(\text{TRT})}(s)ds\}\right] d\nu}{\exp\left\{-\int_0^t h_i^{(\text{PRG})}(\nu)d\nu - \int_0^t h_i^{(\text{TRT})}(\nu)d\nu\right\}},
\end{aligned}
$$

The nested integrals in the above equation do not have a closed-form solution, and can be numerically approximated using the 15-point Gauss-Kronrod rule. Inference can be performed using the following Monto Carlo sampling scheme.[Rizopoulos, 2011]

Web Appendix 3 Model-based weights

The model-based weights of being cases of controls are summarized and explained in Table S1.

Table S1: Intuitive explanation of the model-based weights across all patient groups.

Patient group	Model-based weights of cases	Model-based weights of controls	Note
1a	$\frac{\Pi_i^{\text{PRG}}(T_i^{\text{PRG+}} \mid T_i^{\text{PRG-}}) - \Pi_i^{\text{PRG}}(t \mid T_i^{\text{PRG-}})}{\Pi_i^{\text{PRG}}(T_i^{\text{PRG+}} \mid T_i^{\text{PRG-}})}$<br>progression happens between t and $T_i^{\text{PRG+}}$	0<br>progression cannot happen after $t + \Delta t$	This patient can be excluded (neither a case nor control) if progression happens before t, $\frac{\Pi_i^{\text{PRG}}(t \mid T_i^{\text{PRG-}})}{\Pi_i^{\text{PRG}}(T_i^{\text{PRG+}} \mid T_i^{\text{PRG-}})}$.
1b	$\Pi_i^{\text{PRG}}(T_i^{\text{TRT}} \mid T_i^{\text{PRG-}}) - \Pi_i^{\text{PRG}}(t \mid T_i^{\text{PRG-}})$<br>progression happens between t and T_i^{TRT} but is undetected	0<br>progression cannot happen after $t + \Delta t$, due to early treatment	This patient can either be a case or excluded.
1c	$\Pi_i^{\text{PRG}}(t + \Delta t \mid T_i^{\text{PRG-}}) - \Pi_i^{\text{PRG}}(t \mid T_i^{\text{PRG-}})$<br>progression happens before treatment between t and $t + \Delta t$	$\mathcal{S}_i(t + \Delta t \mid T_i^{\text{PRG-}})$<br>no event happens before $t + \Delta t$	This patient can be excluded if his progression happens before t or treatment happens before progression between t and $t + \Delta t$.
2a	$\frac{\Pi_i^{\text{PRG}}(t + \Delta t \mid T_i^{\text{PRG-}})}{\Pi_i^{\text{PRG}}(T_i^{\text{PRG+}} \mid T_i^{\text{PRG-}})}$<br>progression happens between $T_i^{\text{PRG-}}$ and $t + \Delta t$	$\frac{\Pi_i^{\text{PRG}}(T_i^{\text{PRG+}} \mid T_i^{\text{PRG-}}) - \Pi_i^{\text{PRG}}(t + \Delta t \mid T_i^{\text{PRG-}})}{\Pi_i^{\text{PRG}}(T_i^{\text{PRG+}} \mid T_i^{\text{PRG-}})}$<br>progression happens after $t + \Delta t$	The two model-based weights sum up to one as this patient can only either be a case or a control.
2b	$\Pi_i^{\text{PRG}}(t + \Delta t \mid T_i^{\text{PRG-}})$<br>progression happens between $T_i^{\text{PRG-}}$ and $t + \Delta t$ but is undetected	$1 - \Pi_i^{\text{PRG}}(t + \Delta t \mid T_i^{\text{PRG-}})$<br>all other scenarios as treatment already happens after $t + \Delta t$	The two model-based weights sum up to one as this patient can only either be a case or a control.
2c	$\Pi_i^{\text{PRG}}(t + \Delta t \mid T_i^{\text{PRG-}})$<br>progression happens between $T_i^{\text{PRG-}}$ and $t + \Delta t$ but is undetected	$1 - \Pi_i^{\text{PRG}}(t + \Delta t \mid T_i^{\text{PRG-}})$<br>all other scenarios as treatment cannot happen before $t + \Delta t$	The two model-based weights sum up to one as this patient can only either be a case or a control.
3a	1<br>absolute case	0<br>absolute case	This patient can only be a case.

Continued on next page

Patient group	Model-based weights of cases	Model-based weights of controls	Note
3b	$\Pi_i^{\text{PRG}}(T_i^{\text{TRT}} \mid T_i^{\text{PRG-}})$ progression happens before treatment but is undetected	0 patient cannot be a control	This patient can be excluded if progression does not happen before treatment initiation.
3c	$\Pi_i^{\text{PRG}}(t + \Delta t \mid T_i^{\text{PRG-}})$ progression happens between $T_i^{\text{PRG-}}$ and $t + \Delta t$	$\mathcal{S}_i(t + \Delta t \mid T_i^{\text{PRG-}})$ no event happens before $t + \Delta t$	This patient can be excluded if treatment happens before $t + \Delta t$.
4a, 4b, 4c	0 absolute control	1 absolute control	This patient can only be a control.
5a	$\dfrac{\Pi_i^{\text{PRG}}(t + \Delta t \mid T_i^{\text{PRG-}}) - \Pi_i^{\text{PRG}}(t \mid T_i^{\text{PRG-}})}{\Pi_i^{\text{PRG}}(T_i^{\text{PRG+}} \mid T_i^{\text{PRG-}})}$ progression happens between t and $t + \Delta t$	$\dfrac{\Pi_i^{\text{PRG}}(T_i^{\text{PRG+}} \mid T_i^{\text{PRG-}}) - \Pi_i^{\text{PRG}}(t + \Delta t \mid T_i^{\text{PRG-}})}{\Pi_i^{\text{PRG}}(T_i^{\text{PRG+}} \mid T_i^{\text{PRG-}})}$ progression happens after $t+\Delta t$ (as treatment cannot happen before progression)	This patient can be excluded if progression happens before t, $\dfrac{\Pi_i^{\text{PRG}}(t \mid T_i^{\text{PRG-}})}{\Pi_i^{\text{PRG}}(T_i^{\text{PRG+}} \mid T_i^{\text{PRG-}})}$.
5b	$\Pi_i^{\text{PRG}}(t + \Delta t \mid T_i^{\text{PRG-}}) - \Pi_i^{\text{PRG}}(t \mid T_i^{\text{PRG-}})$ progression happens between t and $t + \Delta t$ but is undetected	$1 - \Pi_i^{\text{PRG}}(t + \Delta t \mid T_i^{\text{PRG-}})$ all other scenarios as treatment already happens after $t + \Delta t$	This patient can be excluded if progression happens before t.
5c	$\Pi_i^{\text{PRG}}(t + \Delta t \mid T_i^{\text{PRG-}}) - \Pi_i^{\text{PRG}}(t \mid T_i^{\text{PRG-}})$ progression happens between t and $t + \Delta t$ but is undetected	$1 - \Pi_i^{\text{PRG}}(t + \Delta t \mid T_i^{\text{PRG-}})$ all other scenarios as treatment cannot happen before $t + \Delta t$	This patient can be excluded if progression happens before t.

Web Appendix 4 Simulation

Web Appendix 4.1 Simulation setting

We simulated data based on the parameters from the ICJM (see Section 3 of the manuscript) fitted on the Canary PASS data. The patients in this study were scheduled to undergo biopsies in months 12, 24 and afterwards biennially and to have PSA measurements taken every three months. The ICJM had the following structure:

$$
\begin{aligned}
\log_2(\text{PSA}_j + 1)(t) &= m_{\text{PSA},j}(t) + \epsilon_j(t), \\
m_{\text{PSA},j}(t) &= \beta_0 + u_{0j} + \sum_{p=1}^{3}(\beta_p + u_{pj})\mathcal{C}_j^{(p)}(t) + \beta_4(\text{Age}_j - 62), \\
h_j^{(k)}\left\{t \mid \boldsymbol{\mathcal{M}}_{\text{PSA},j}(t)\right\} &= h_0^{(k)}(t)\exp\left[\gamma_k \text{density}_j + f\left\{\boldsymbol{\mathcal{M}}_{\text{PSA},j}(t), \boldsymbol{\alpha}_k\right\}\right],
\end{aligned}
$$

where $\mathcal{C}(t)$is the design matrix for the natural cubic splines (with three degrees of freedom) for time t; Age_j and density_j refer to the patient's age and PSA density at the start of active surveillance. Baseline age was centered by subtracting the median age (62 years) for computational reasons. Both the expected value of PSA and the change in expected PSA over the previous year (where extrapolation was conducted for time points earlier than year one) were included as covariates in the time-to-event component, i.e.,

$$
f\left\{\boldsymbol{\mathcal{M}}_{\text{PSA},j}(t), \boldsymbol{\alpha}_k\right\} = \alpha_{1k,\text{PSA}} m_{\text{PSA},j}(t) + \alpha_{2k,\text{PSA}}\Big\{m_{\text{PSA},j}(t) - m_{\text{PSA},j}(t-1)\Big\}.
$$

The residuals of the longitudinal component were assumed to follow a Student's t distribution with three degrees of freedom [Tomer et al., 2022],

$$
\epsilon_j(t) \sim t(\frac{1}{\tau_\epsilon}, 3),
$$

with

$$
\tau_\epsilon \sim \text{Gamma}(0.01, 0.01).
$$

The prior distributions for the regression coefficients were specified as vague normal distributions,

$$
\begin{aligned}
\beta &\sim \mathcal{N}(0, 100), \\
\gamma_k &\sim \mathcal{N}(0, 100), \\
\alpha_{1k,\text{PSA}}, \alpha_{2k,\text{PSA}} &\sim \mathcal{N}(0, 100),
\end{aligned}
$$

and the variance-covariance matrix of the random effects, $\boldsymbol{\Omega}$, to follow an inverse-Wishart distribution,

$$
\boldsymbol{\Omega} \sim \mathcal{IW}(n_u + 1, \frac{4}{\tau_u}),
$$

with

$$
\tau_u \sim \text{Gamma}(0.5, 0.01),
$$

where n_u is the number of parameters in the variance-covariance matrix of the random effects.

The model was implemented in JAGS [Plummer, 2003] and run for 10000 iterations, using a thinning interval of 10, in each of three MCMC chains.

The resulting posterior means used for simulation were

$$\boldsymbol{\beta} = [2.34, 0.28, 0.61, 0.95, 0.02]^\top,$$

$$\boldsymbol{\Omega} = \begin{bmatrix} 0.48 & -0.04 & -0.07 & 0.02 \\ -0.04 & 0.77 & 0.46 & -0.04 \\ -0.07 & 0.46 & 1.37 & 1.36 \\ 0.02 & -0.04 & 1.36 & 2.54 \end{bmatrix},$$

$$\tau_\epsilon = 47.40,$$

$$\boldsymbol{\gamma}_{h_0} = \begin{bmatrix} -6.78 & -5.76 \\ -4.72 & -4.99 \\ -2.84 & -4.43 \\ -1.65 & -4.26 \\ -1.54 & -4.36 \\ -1.79 & -4.47 \\ -1.85 & -4.60 \\ -1.75 & -4.69 \\ -1.85 & -4.78 \\ -2.04 & -4.92 \\ -2.18 & -5.08 \\ -2.32 & -5.21 \end{bmatrix},$$

$$\boldsymbol{\gamma} = [0.50, 0.23],$$

$$\boldsymbol{\alpha} = \begin{bmatrix} 0.13 & 0.42 \\ 3.01 & 2.62 \end{bmatrix}.$$

The resulting simulated data matched the observed data well with regard to the rates of cancer progression, early treatment initiation and censoring (Table S2).

Table S2: Summary of event proportions in the simulated training datasets compared to the observed data.

**Events**	**Simulated data**† (%)	**Observed data** (%)
Cancer progression	22.35	21.97
Treatment	9.18	10.44
Censoring	68.47	67.59

†: the average proportions overall 200 datasets are presented.

Web Appendix 4.2 Misspecification of the linear model

We randomly select 8 patients from the first evaluation (on the second simulated dataset) and show the fitted trajectory of PSA (transformed in $\log_2(\text{PSA} + 1)$) resulting from the models assuming a linear trajectory versus natural cubic splines (with 3 degrees of freedom, i.e., the correctly-specified model). The trajectories are shown in Figure S1.

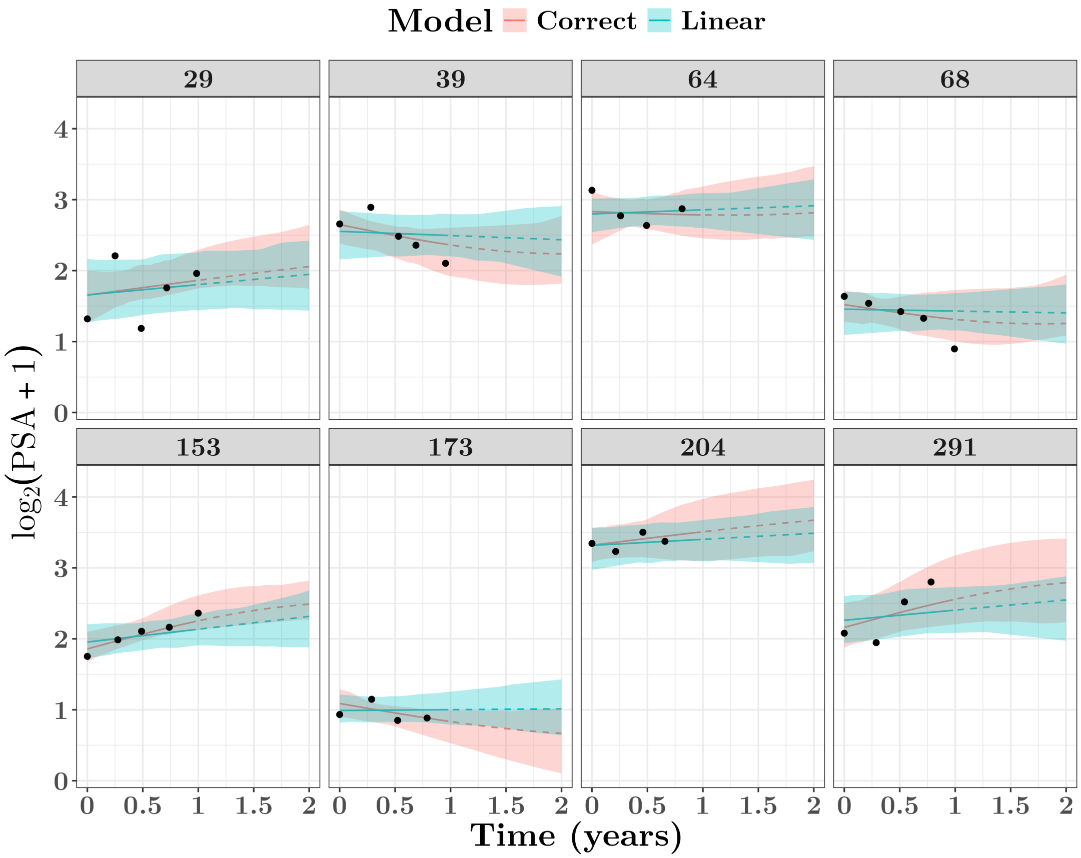

Figure S1: The observed trajectories (in black points), fitted trajectories (with 95% credible interval presented in ribbons) assuming a linear effect of time (in blue, as in the linear models) and natural cubic splines with 3 degrees of freedom (in red, as in the correctly-specified models) of 8 randomly selected patients from the simulated datasets. The fit for the time after year 1 (indicated by dashed lines) is included to provide a better picture of the degree of non-linearity of the spline fit.

Web Appendix 4.3 Calculation of EPCE in the scenario without censoring

In the manuscript, we showed the estimation of the EPCE using the model-based risks, in the setting with interval censoring and competing risks. For the simulation study, the reference EPCE is calculated with the true event times, thus without interval cenosring. It can be estimated by

$$\widehat{\text{EPCE}}^{\text{PRG}}_{\text{ref}}(t+\Delta t, t) = \frac{1}{n_t} \sum_{i:\min(T_i^{\text{PRG}*}, T_i^{\text{TRT}*}) \geq t} -\log\left[p\{\tilde{T}_i, \tilde{\delta}_i^{\text{ref}(1)}, \tilde{\delta}_i^{\text{ref}(2)} \mid T_i^{\text{PRG}*} \geq t, T_i^{\text{PRG}*} < T_i^{\text{TRT}*}, \boldsymbol{\mathcal{Y}}_i(t), \boldsymbol{\mathcal{D}}_n\}\right],$$

where $\tilde{T}_i = \min(T_i^{\text{PRG}*}, t+\Delta t)$, $\tilde{\delta}_i^{\text{ref}(1)} = I\left(T_i^{\text{PRG}*} \geq t, T_i^{\text{PRG}*} < t+\Delta t, T_i^{\text{PRG}*} < T_i^{\text{TRT}*}\right)$, and $\tilde{\delta}_i^{\text{ref}(2)} = I\left\{T_i^{\text{PRG}*} \geq \min(T_i^{\text{TRT}*}, t+\Delta t)\right\}$. The term in the log function can be estimated by

$$p\{\tilde{T}_i, \tilde{\delta}_i^{\text{ref}(1)}, \tilde{\delta}_i^{\text{ref}(2)} \mid T_i^{\text{PRG}*} \geq t, T_i^{\text{PRG}*} < T_i^{\text{TRT}*}, \boldsymbol{\mathcal{Y}}_i(t), \boldsymbol{\mathcal{D}}_n\} = \log\left[\tilde{\delta}_i^{(1)} \mathcal{F}_1^{\text{ref}} + \tilde{\delta}_i^{(2)} \mathcal{F}_2^{\text{ref}}\right],$$

where the first factor $\mathcal{F}_1^{\text{ref}} = \Pr\{T_i^{\text{PRG}*} \leq \tilde{T}_i \mid T_i^{\text{PRG}*} \geq t, T_i^{\text{PRG}*} < T_i^{\text{TRT}*}, \boldsymbol{\mathcal{Y}}_i(t), \boldsymbol{\mathcal{D}}_n\}$ is the cumulative incidence function of progression until $\tilde{T}_i$, and the second factor $\mathcal{F}_2^{\text{ref}} = \frac{\Pr\{T_i^* \geq \tilde{T}_i \mid \boldsymbol{\mathcal{Y}}_i(t), \boldsymbol{\mathcal{D}}_n\}}{\Pr\{T_i^* \geq t \mid \boldsymbol{\mathcal{Y}}_i(t), \boldsymbol{\mathcal{D}}_n\}}$ is the overall survival probability of the patient surviving after $\tilde{T}_i$ conditional on that he did not experience either of the events until t.

References

Kerri Beckmann, Declan Cahill, Christian Brown, Mieke Van Hemelrijck, and Netty Kinsella. Understanding reasons for non-adherence to active surveillance for low-intermediate risk prostate cancer. *Translational Andrology and Urology*, 10(6), 2021. ISSN 2223-4691. URL `https://tau.amegroups.org/article/view/64833`.

Martyn Plummer. Jags: A program for analysis of bayesian graphical models using gibbs sampling. *3rd International Workshop on Distributed Statistical Computing (DSC 2003); Vienna, Austria*, 124, 04 2003.

Dimitris Rizopoulos. Dynamic predictions and prospective accuracy in joint models for longitudinal and time-to-event data. *Biometrics*, 67(3):819–829, 2011.

Anirudh Tomer, Daan Nieboer, Monique J. Roobol, Ewout W. Steyerberg, and Dimitris Rizopoulos. Shared decision making of burdensome surveillance tests using personalized schedules and their burden and benefit. *Statistics in Medicine*, 41(12):2115–2131, 2022. doi: https://doi.org/10.1002/sim.9347.

Jeffrey J. Tosoian, H. Ballentine Carter, Abbey Lepor, and Stacy Loeb. Active surveillance for prostate cancer: current evidence and contemporary state of practice. *Nature Reviews Urology*, 13(4):205–215, Apr 2016. ISSN 1759-4820. doi: 10.1038/nrurol.2016.45.

Zhenwei Yang, Dimitris Rizopoulos, Eveline A. M. Heijnsdijk, Lisa F. Newcomb, and Nicole S. Erler. Personalized biopsy schedules using an interval-censored cause-specific joint model. *Statistics in Medicine*, 44(10-12):e70134, 2025. doi: 10.1002/sim.70134.
