# Supplementary material for: Time‐Dependent Predictive Accuracy Metrics in the Context of Interval Censoring and Competing Risks
Source: Biom J. 2026 Jan 5;68(1):e70108. doi: 10.1002/bimj.70108 (PMC12766878; doi:10.1002/bimj.70108)
Supplement: Supplementary file 2 — Supporting File: bimj70108‐sup‐0002‐Datacode.zip. [file BIMJ-68-e70108-s001.zip › Supplement code/Predicitve-accuracy-metrics-main/README.html]

README


# Title: Time-dependent Predictive Accuracy Metrics in the Context of Interval Censoring and Competing Risks

**Author**: Zhenwei Yang

**Affiliation**: Department of Biostatistics, Erasmus
Medical Center

**DOI**: [under review]

---

## Content

- Predicitve-accuracy-metrics.Rproj
- Data
- Rscript
- Output
- img

---

### Data

| File name | Description |
| --- | --- |
| ICJM1.RData | the ICJM1 results |
| CV | the folder containing model results of ICJM1 based on 5-fold cross-validation |
| Simulation Datasets | Simulated datasets used in simulation including PASS and random schdules |

### Rscript

- Data analysis

| File name | Description |
| --- | --- |
| evaluation metric\_PASS\_CV.R | the script to evaluate the ICJM1 based on 5-fold cross-validation |

- Function

| File name | Description |
| --- | --- |
| dynpred\_corspe.R | the function used to generate predictions from the correctly-specified ICJM1 |
| dynpred\_linear.R | the function used to generate predictions from the ICJM1 with linear PSA |
| dynpred\_nocovar.R | the function used to generate predictions from the ICJM1 ignoring baseline covariate PSA density |
| function.R | help and auxiliary functions |
| ICJM\_correctlyspecify.R | the function used to fit the correctly-specified ICJM1 |
| ICJM\_linear.R | the function used to fit the ICJM1 with linear PSA |
| ICJM\_nocovar.R | the function used to fit the ICJM1 ignoring baseline covariate PSA density |
| time dependent evaluation metrics.R | the function used for model evaluation based on AUC, Brier score and EPCE |
| time dependent true EPCE.R | the function used to calculate the EPCE in absence of censoring |

- Simulation

| File name | Description |
| --- | --- |
| Seed | including the seeds to generate the data & fit the model, the seeds for individual’s personalized scheduling, and model test |
| Dataset generation.R | training and test sets generation based on three models and four scheduling scenarios |
| MM\_fit.R | fit the longitudinal submodel used as an initial values in the joint model estimation |
| Model Estimation.R | model fit based on 200 simulated training sets according to the model specification and scheduling scenarios |
| Evaluation.R | evaluate the simulated models based on AUC, Brier score and EPCE |
| Evaluation\_naive.R | evaluate the simulated models based on AUC, Brier score and EPCE using the naive approach (ignoring censoring) |
| Evaluation\_true.R | evaluate the simulated models based on reference AUC, Brier score and EPCE in absence of censoring |

- Tables&figures

| File name | Description |
| --- | --- |
| Figure tables\_main.R | draw the plots and tables in the manuscript |
| Figure tables\_supplement.R | draw the plots and tables in the supplement |

### Output

- Data analysis

| File name | Description |
| --- | --- |
| AccMet\_CV\_1.RData | the model evaluation results (AUC, Brier score and EPCE) of the first fold version of the ICJM1 |
| AccMet\_CV\_2.RData | the model evaluation results (AUC, Brier score and EPCE) of the second fold version of the ICJM1 |
| AccMet\_CV\_3.RData | the model evaluation results (AUC, Brier score and EPCE) of the third fold version of the ICJM1 |
| AccMet\_CV\_4.RData | the model evaluation results (AUC, Brier score and EPCE) of the fourth fold version of the ICJM1 |
| AccMet\_CV\_5.RData | the model evaluation results (AUC, Brier score and EPCE) of the fifth fold version of the ICJM1 |

- Simulation Evaluation
- Simulation Models

| File name | Description |
| --- | --- |
| Correctly specified models | correctly-specified joint models |
| Fitted mixed model | mixed model part for the initials for fitting the joint models |
| Linear models | joint models assuming linear PSA |
| Linear models | joint models omiting PSA density |

- Simulation Storage

| File name | Description |
| --- | --- |
| corspe | detailed predictions for correctly-specified joint model to visualized PSA trajectories |
| linear | detailed predictions for joint model assuming linear PSA to visualized PSA trajectories |

- Tables&figures

| File name | Description |
| --- | --- |
| Main text | all figures used in the manuscripts |
| Supplementary | all figures used in the supplementary materials |

---

## Reproducibility

### Package dependencies

- rajgs
- mcmcplots
- GLMMadaptive
- ggplot2
- tidyverse
- splines
- future
- mcmcse
- mvtnorm
- JMbayes2
- JMbayes
- MASS
- doParallel
- truncnorm
- Matrix
- latex2exp
- cowplot
- PCaASSim (this can be downloaded via:
  `devtools::install_github("https://github.com/ZhenweiYang96/PCaASSim")`)

> [!Note] - Please make sure to install above-mentioned packages by
> `install.packages()` before running the R code

### Instructions for reproducing the results

#### Section 3 - Application

- The evaluation metrics for the Canary PASS data (with cross
  validation) can be derived from this path: */R script/Data
  analysis/evaluation metric\_PASS\_CV.R* (In the process, there will be
  some saved results, e.g., the model results for each fold stored under
  */Data/CV/ICJM\_CV\_1.RData* to
  */Data/CV/ICJM\_CV\_5.RData*).

> [!Note] - The original Canary PASS data (*/Data/pass\_id.RData*
> and */Data/pass.RData*) is not publically sharable and thus does
> not present in this repository.

#### Section 4 - Simulation

1. Data generation: run */R script/Simulation/Data
   Generation.R* to generate \(200 \times
   4\) datasets. There are four types of scnearios: the standard
   PASS schedule (stored under */Data/Simulation Datasets/*), three
   random schedules (stored under */Data/Simulation
   Datasets/random\_3\_10/*, */Data/Simulation
   Datasets/random\_3\_40/* and */Data/Simulation
   Datasets/random\_10\_20/*)
2. Preparation for model fitting: run */R
   script/Simulation/MM\_fit.R*.
3. Model fitting for each simulated dataset: run */R
   script/Simulation/Model Estimation.R*. For each dataset, three
   models were fitted: correctly-specified model stored under
   */Output/Simulation Models/Correctly specified models/*, model
   with linear PSA stored under */Output/Simulation Models/Linear
   models/*, and the model ignoring the baseline covariate PSA density
   stored under */Output/Simulation Models/No covariate
   models/*.
4. Model evaluating (two proposed approaches in the manuscript): run
   */R script/Simulation/Evaluation.R*. Results should be stored
   under */Output/Simulation Evaluation/*, and are needed for the
   plots.
5. Model evaluating (the naive approach in the manuscript): run
   */R script/Simulation/Evaluation\_naive.R*. Results should be
   stored under */Output/Simulation Evaluation/Observed/*, and are
   needed for the plots.
6. Model evaluating (the reference in the manuscript): run */R
   script/Simulation/Evaluation\_true.R*. Results should be stored under
   */Output/Simulation Evaluation/True linear/*,
   */Output/Simulation Evaluation/True models/* and
   */Output/Simulation Evaluation/True no cov/*, and are needed for
   the plots.

#### Figures and Tables in the manuscript and supplements

- run */R script/Tables & figures/Figure tables\_main.R*
  for all the tables and figures in manuscript. This file uses the all the
  results from the previous steps which should be stored under
  */Output/*. The figures are stored under */Output/Tables &
  figures/Main text/*.
- run */R script/Tables & figures/Figure
  tables\_supplement.R* for all the tables and figures in supplements.
  This file uses the all the results from the previous steps which should
  be stored under */Output/*. The figures are stored under
  */Output/Tables & figures/Supplementary/*.

> [!Note] - Plotting the figures needs the `LM Roman 10`
> font. Please install this font in your system. If that is not
> applicable, please comment out the code line
> `theme(..., text = element_text(..., family = "LM Roman 10"))`
> to make the codes work seamlessly!

---

## Session Info

```
R version 4.3.3 (2024-02-29)
Platform: x86_64-apple-darwin20 (64-bit)
Running under: macOS 15.4.1

Matrix products: default
BLAS:   /System/Library/Frameworks/Accelerate.framework/Versions/A/Frameworks/vecLib.framework/Versions/A/libBLAS.dylib 
LAPACK: /Library/Frameworks/R.framework/Versions/4.3-x86_64/Resources/lib/libRlapack.dylib;  LAPACK version 3.11.0

locale:
[1] en_US.UTF-8/en_US.UTF-8/en_US.UTF-8/C/en_US.UTF-8/en_US.UTF-8

time zone: Europe/Amsterdam
tzcode source: internal

attached base packages:
[1] splines   parallel  stats     graphics  grDevices utils     datasets  methods   base     

other attached packages:
 [1] mcmcse_1.5-0       rjags_4-15         coda_0.19-4.1      GLMMadaptive_0.9-1
 [5] future_1.33.2      survival_3.5-8     PCaASSim_0.1.0     cowplot_1.1.3     
 [9] lubridate_1.9.3    forcats_1.0.0      stringr_1.5.1      dplyr_1.1.4       
[13] purrr_1.0.2        readr_2.1.5        tidyr_1.3.1        tibble_3.2.1      
[17] ggplot2_3.5.0      tidyverse_2.0.0   

loaded via a namespace (and not attached):
 [1] gridExtra_2.3      remotes_2.5.0      inline_0.3.19      testthat_3.2.1.1  
 [5] rlang_1.1.3        magrittr_2.0.3     matrixStats_1.3.0  compiler_4.3.3    
 [9] loo_2.7.0          fftwtools_0.9-11   vctrs_0.6.5        profvis_0.3.8     
[13] pkgconfig_2.0.3    fastmap_1.1.1      backports_1.4.1    ellipsis_0.3.2    
[17] utf8_1.2.4         promises_1.3.0     rmarkdown_2.26     sessioninfo_1.2.2 
[21] tzdb_0.4.0         xfun_0.51          cachem_1.0.8       jsonlite_1.8.8    
[25] later_1.3.2        cluster_2.1.6      R6_2.5.1           stringi_1.8.3     
[29] StanHeaders_2.32.7 parallelly_1.37.1  pkgload_1.3.4      rpart_4.1.23      
[33] brio_1.1.5         Rcpp_1.0.12        rstan_2.32.6       iterators_1.0.14  
[37] knitr_1.45         usethis_2.2.3      base64enc_0.1-3    httpuv_1.6.15     
[41] Matrix_1.6-5       nnet_7.3-19        timechange_0.3.0   tidyselect_1.2.1  
[45] rstudioapi_0.16.0  doParallel_1.0.17  codetools_0.2-19   miniUI_0.1.1.1    
[49] listenv_0.9.1      pkgbuild_1.4.4     lattice_0.22-5     shiny_1.8.1.1     
[53] withr_3.0.0        evaluate_0.23      foreign_0.8-86     RcppParallel_5.1.7
[57] urlchecker_1.0.1   pillar_1.9.0       JMbayes_0.8-85     checkmate_2.3.1   
[61] foreach_1.5.2      stats4_4.3.3       ellipse_0.5.0      generics_0.1.3    
[65] mathjaxr_1.6-0     truncnorm_1.0-9    hms_1.1.3          munsell_0.5.1     
[69] scales_1.3.0       globals_0.16.3     xtable_1.8-4       glue_1.7.0        
[73] Hmisc_5.1-2        tools_4.3.3        data.table_1.15.4  mvtnorm_1.2-4     
[77] fs_1.6.3           grid_4.3.3         QuickJSR_1.1.3     devtools_2.4.5    
[81] colorspace_2.1-0   nlme_3.1-164       htmlTable_2.4.2    Formula_1.2-5     
[85] cli_3.6.2          fansi_1.0.6        gtable_0.3.4       digest_0.6.35     
[89] htmlwidgets_1.6.4  memoise_2.0.1      htmltools_0.5.8.1  lifecycle_1.0.4   
[93] jagsUI_1.6.2       mime_0.12          MASS_7.3-60.0.1
```

---

## Poster

- The corresponding poster presented in ISCB 2024:
