# Supplementary figures and images for: Time‐Dependent Predictive Accuracy Metrics in the Context of Interval Censoring and Competing Risks
Source: Biom J. 2026 Jan 5;68(1):e70108. doi: 10.1002/bimj.70108 (PMC12766878; doi:10.1002/bimj.70108)

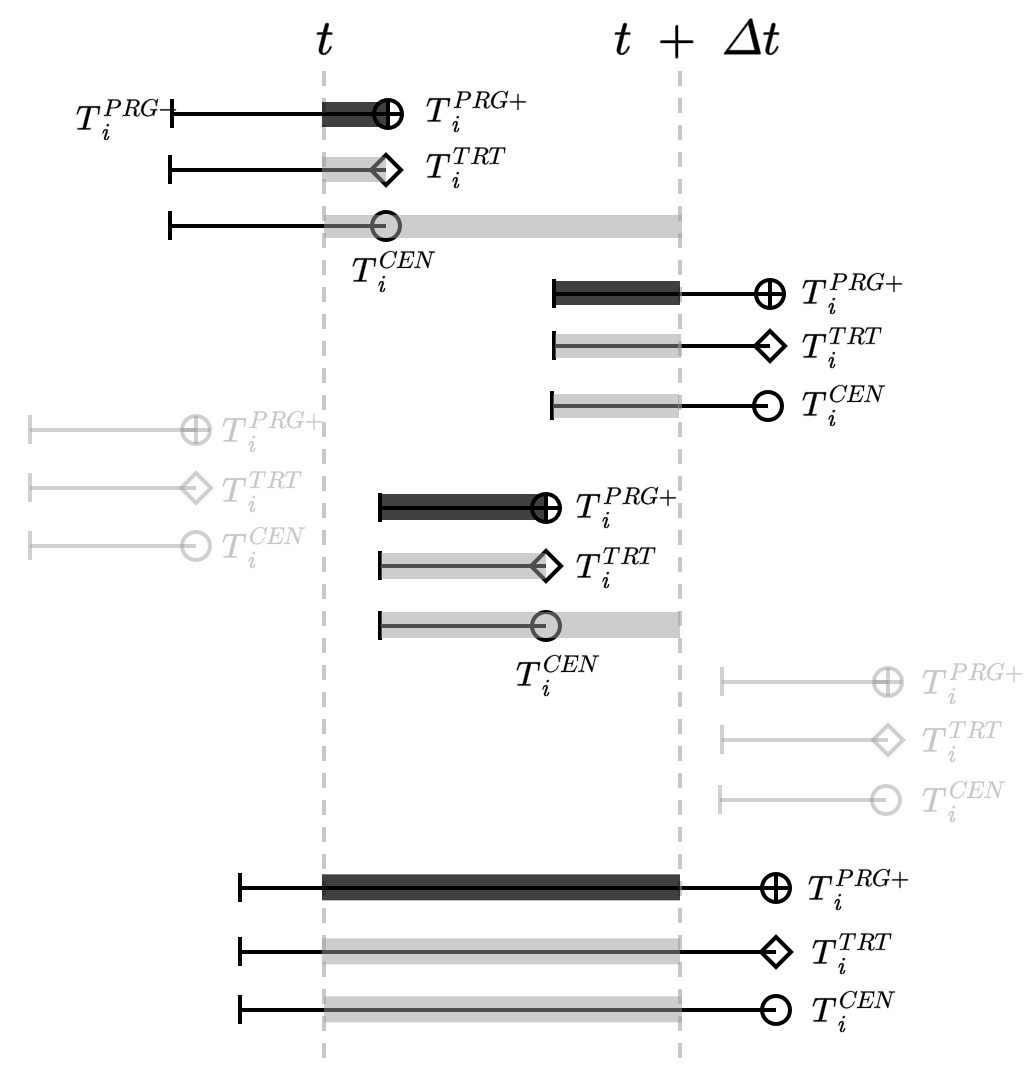

Supplement: Supplementary file 2 — Supporting File: bimj70108‐sup‐0002‐Datacode.zip. [file BIMJ-68-e70108-s001.zip › Supplement code/Predicitve-accuracy-metrics-main/Output/Tables & figures/Main text/sensitivity weights.png]

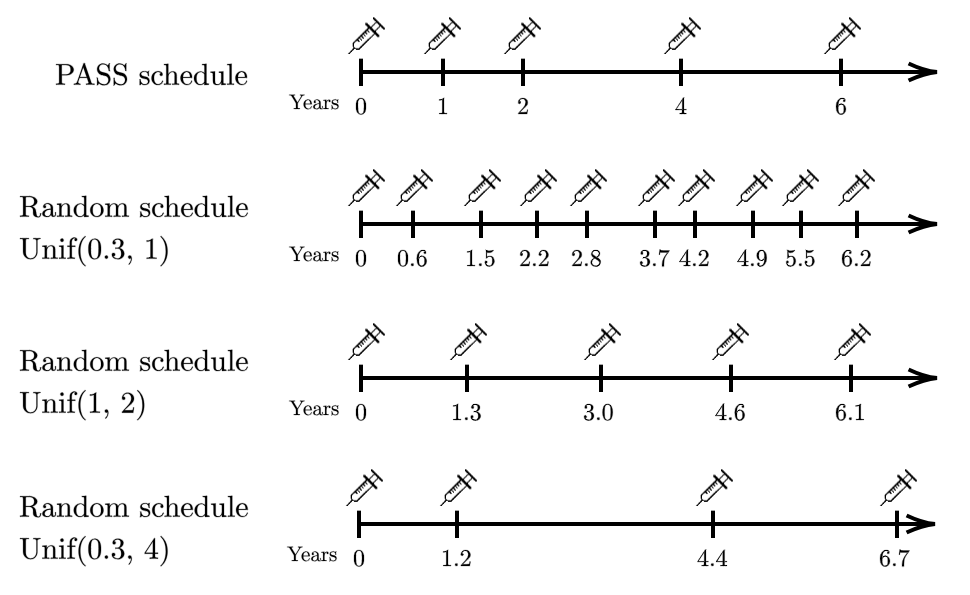

Supplement: Supplementary file 2 — Supporting File: bimj70108‐sup‐0002‐Datacode.zip. [file BIMJ-68-e70108-s001.zip › Supplement code/Predicitve-accuracy-metrics-main/Output/Tables & figures/Main text/simulation scenario examples.png]

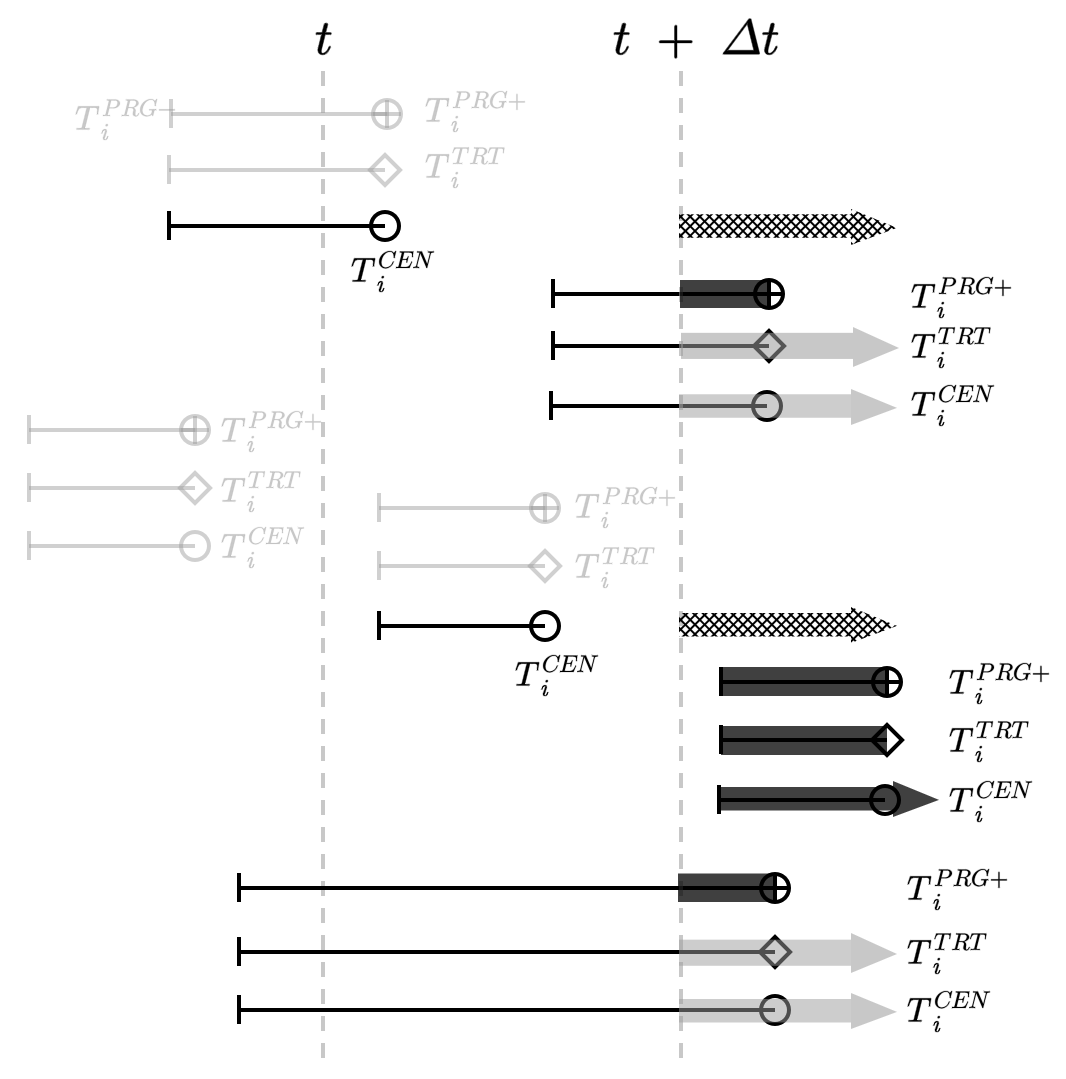

Supplement: Supplementary file 2 — Supporting File: bimj70108‐sup‐0002‐Datacode.zip. [file BIMJ-68-e70108-s001.zip › Supplement code/Predicitve-accuracy-metrics-main/Output/Tables & figures/Main text/specificity weights.png]
